# Pharmacological considerations for prescribing of small molecule inhibitors in patients with non-small cell lung cancer

**DOI:** 10.1016/j.eclinm.2026.104199

**Published:** 2026-09-15

**Authors:** Lotte M.G. Hulskotte, G.D. Marijn Veerman, Daan A.C. Lanser, Anna K.L. Reyners, Ron H.N. van Schaik, Anne-Marie C. Dingemans, Katja Taxis, Ron H.J. Mathijssen, Frank G.A. Jansman

**Affiliations:** a Unit of PharmacoTherapy, -Epidemiology &-Economics, Groningen Research Institute of Pharmacy (GRIP), University of Groningen, Groningen, the Netherlands; b Department of Clinical Pharmacy, Deventer Teaching Hospital, Deventer, the Netherlands; c Department of Pulmonary Medicine, Erasmus MC Cancer Institute, Erasmus University Medical Center, Rotterdam, the Netherlands; d Department of Medical Oncology, Erasmus MC Cancer Institute, Erasmus University Medical Center, Rotterdam, the Netherlands; e Department of Clinical Chemistry, Erasmus University Medical Center, Rotterdam, the Netherlands; f Department of Medical Oncology, University of Groningen, University Medical Center Groningen, Groningen, the Netherlands

**Keywords:** Non-small cell lung cancer, Small molecule inhibitors, Drug interactions, Pharmacology, Adverse effects, Pharmacokinetics

## Abstract

Small molecule inhibitors (SMIs) have become the cornerstone of treatment for patients with oncogene-driven non-small cell lung cancer (NSCLC), substantially improving clinical outcomes. Over the past decade, numerous agents targeting distinct oncogenic drives have been developed, each exhibiting unique pharmacokinetic and pharmacodynamic profiles that complicate clinical prescribing. This review highlights principal drug-interactions affecting SMI exposure, including concomitant food intake, gastric acid suppressants, modulators of cytochrome P450 (CYP) enzymes and drug transporters. Awareness of these factors may help prevent early treatment failure or severe adverse events due to under- or overexposure. Finally, the most prevalent and clinically relevant toxicities are discussed, including corrected QT interval (QTc) prolongation, pneumonitis, mucositis, weight gain, pyrexia, and ocular-, hepatic-, and neuro-toxicity. In line with recommendations from the US Food and Drug Administration (FDA) and the European Medicines Agency (EMA), this review which is based on extensive search of the scientific literature provides evidence-based practical guidance for prescribing SMIs in clinical practice.

**Search strategy and selection criteria**

We conducted a comprehensive review of the existing scientific literature to identify known or suspected drug–drug and food–drug interactions of small molecule kinase inhibitors (SMIs) approved for the use of non-small cell lung cancer (NSCLC), by the US Food and Drug Administration (FDA) and the European Medicines Agency (EMA) and commonly prescribed conventional medications. Furthermore, we examined published evidence on pharmacodynamic adverse effects of FDA- and EMA-approved SMIs, focusing on corrected QT interval (QTc) interval prolongation, pneumonitis, weight gain, hepatic toxicity, neurotoxicity, ocular toxicity, and non-infectious pyrexia. Relevant studies were identified through systematic searches of PubMed (MESH terms) and Embase databases. The initial search was performed on October 13, 2025, periodically updated during manuscript preparation, and finalised on July 8, 2026. The detailed search strategies for PubMed and Embase are provided in the Supplementary. Additional data were extracted from publicly available regulatory documents of SMIs approved by the FDA or EMA as of July 8, 2026. To avoid duplication of evidence, data from original pharmacokinetic studies or pivotal clinical trials that were already fully represented in regulatory documents were not included separately. Studies solely based on pharmacovigilance database analyses (*e.g.,* FDA Adverse Event Reporting System) were excluded from this review. Eligibility was restricted to SMIs approved by the FDA and/or EMA for the treatment of NSCLC. Only articles published in English were considered. The SMIs included are adagrasib, afatinib, alectinib, aumolertinib, binimetinib, brigatinib, capmatinib, ceritinib, crizotinib, dabrafenib, dacomitinib, encorafenib, ensartinib, entrectinib, erlotinib, gefitinib, larotrectinib, lazertinib, lorlatinib, osimertinib, pralsetinib, repotrectinib, selpercatinib, sevabertinib, sotorasib, sunvozertinib, taletrectinib, tepotinib, trametinib and zongertinib.

A pragmatic hierarchy of evidence was applied to clinical recommendations. The supporting evidence was classified as follows: (1) high-level evidence: pharmacokinetic studies, systematic reviews, meta-analyses, and pooled analyses of prospective clinical trials, providing direct support for the recommendation; (2) moderate-level evidence: phase 1/2 clinical trials, non-randomised prospective studies, observational studies, real-world studies, retrospective cohort studies, and post-hoc analyses; (3) Regulatory documents: FDA or EMA labels, product information, assessment reports and review files; (4) expert-based evidence: expert consensus statements, position papers, narrative reviews, and other publications in which recommendations are primarily based on expert opinion and interpretation of the available evidence rather than direct clinical evidence; (5) authors’ expert opinion: formulated by the authors based on review and interpretation of the available evidence when no direct supporting evidence or external expert guidance is available. Case reports were excluded from the hierarchy of evidence, because they were not included in the evidence underpinning the recommendations. Recommendations may be supported by one or more evidence categories, reflecting the different sources that contributed to the final recommendation.

## Introduction

Small molecule inhibitors (SMIs) have fundamentally transformed the treatment landscape of non-small cell lung cancer (NSCLC). These agents are now integral to the clinical management of oncogene-driven advanced NSCLC.^1^, ^2^, ^3^, ^4^ Their different pharmacokinetic profiles contribute to inter-agent variability in both on- and off-target toxicities, as well as the potential for drug interactions affecting SMI exposure, including alterations in gastric pH, modulation of drug transporters and cytochrome P450 (CYP) enzymes, and food intake.^5^, ^6^, ^7^, ^8^ The growing number of approved SMIs increases the clinical complexity of their prescription. Although several reviews have discussed pharmacokinetic interactions and toxicities of individual SMIs or specific drug classes, the field continues to evolve rapidly, requiring ongoing integration of new evidence and clinical insights into contemporary treatment recommendations. This Review therefore provides an updated and clinically focused overview of currently approved SMIs for NSCLC.

In this Review, we examine the principal mechanisms of drug interactions affecting the pharmacokinetics of US Food and Drug Administration (FDA) and European Medicines Agency (EMA) approved SMIs to treat NSCLC, focusing on transporters, CYP-enzymes, acid-reducing agents, and food. We further highlight specific pharmacodynamic effects associated with these SMIs, resulting in clinically significant complications. Finally, we provide a practical up-to-date overview of published studies to support informed individual decision-making and treatment management with SMIs in clinical practice.

## Pharmacokinetic effects

Pharmacokinetic interactions involving SMIs are primarily governed by processes affecting absorption, metabolism, and excretion, as these mechanisms directly influence systemic drug exposure by altering bioavailability and clearance.^7^^,^^9^ Interactions at the distribution level are considered not clinically significant for SMIs, as the rapid clearance of increased unbound drug fractions generally minimises their impact.^10^

**Fig. 1:**
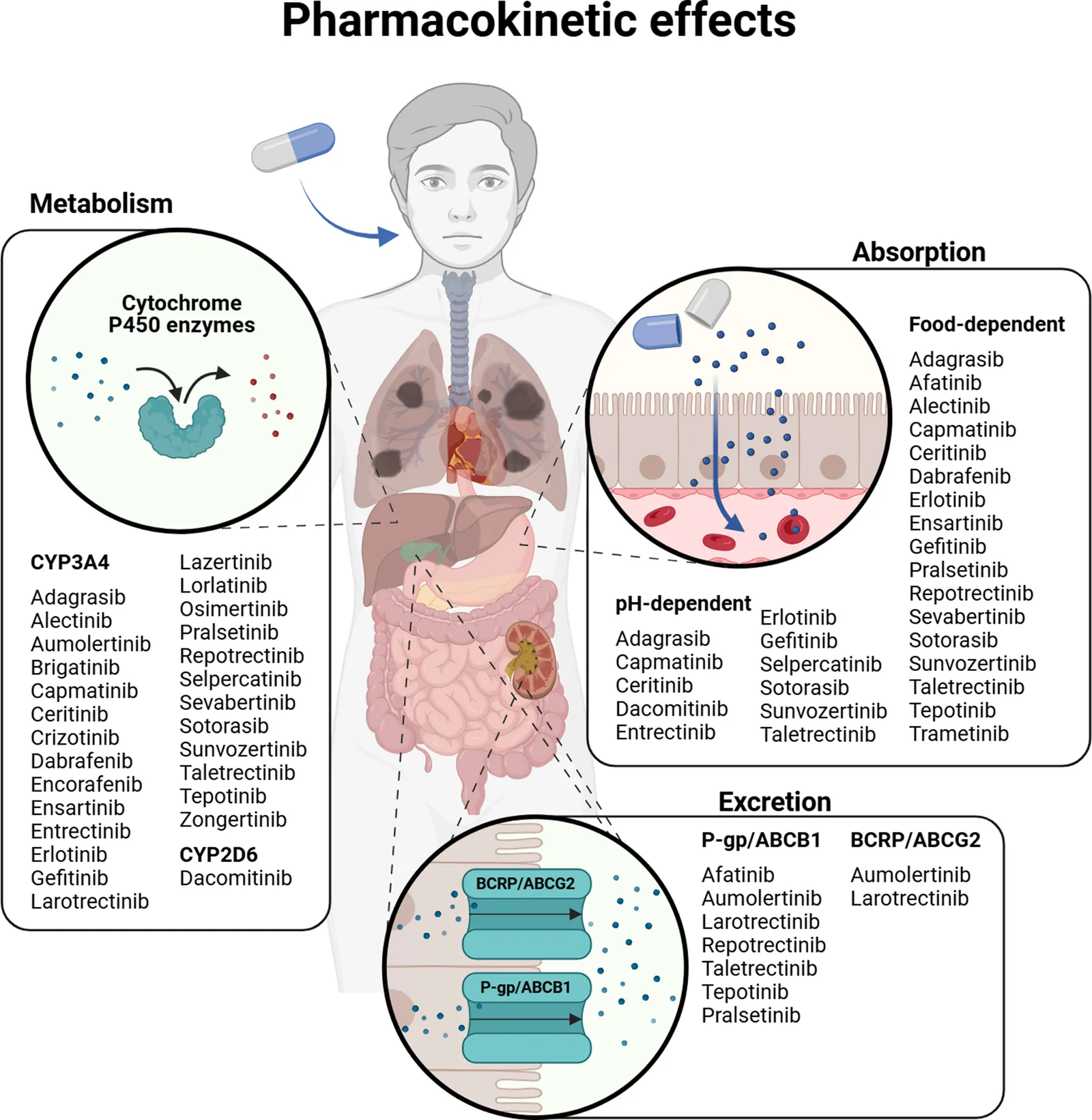
**A comparative overview of clinically relevant pharmacokinetic effects affecting the pharmacokinetics of small molecule inhibitors used in the treatment of non-small cell lung cancer, focusing on cytochrome P450 (CYP)-enzymes, acid reducing agents, and excretion**.

SMIs are often weakly basic compounds with a lipophilic profile, making their bioavailability susceptible to various physiological and pharmacological factors.^11^ Elevation of gastric pH, as observed with the administration of acid-reducing agents, decreases the bioavailability of weakly basic SMIs by shifting the ionisation equilibrium toward the non-ionised, les aqueous-soluble form, which complicates the dissolution process.^11^ By contrast, while food intake usually temporarily increases gastric pH, concurrent intake with (high-fat) meals often enhances bioavailability of several SMIs.^8^^,^^12^ This effect may be driven by delayed gastric and intestinal transit, increased gastric fluid volume, enhanced splanchnic blood and bile flow, collectively creating a lipophilic environment that promotes solubilisation and improves gastrointestinal absorption.^8^^,^^12^ Moreover, the absorption of SMIs that are substrates of efflux transporters such as P-glycoprotein (*i.e.,* ABCB1) and breast cancer resistance protein (*i.e.,* ABCG2) can be influenced by transporter-inducing or -inhibiting co-medication. For instance, induction of these transporters enhances efflux into the intestinal lumen, thereby reducing systemic absorption. Conversely, inhibition decreases efflux, resulting in enhanced drug absorption.^7^^,^^13^ As most SMIs are primarily metabolised via CYP450 iso-enzymes, inducers, inhibitors and genetic polymorphisms of CYP-enzymes may accelerate or reduce metabolic clearance, resulting in decreased or increased systemic exposure, respectively.^13^, ^14^, ^15^ In particular, most SMIs are metabolised by CYP3A (Fig. 1), which is for instance inhibited by grapefruit, thereby increasing the exposure to CYP3A-metabolised SMIs.^16^^,^^17^ The CYP3A4-mediated metabolism is often associated with P-glycoprotein involvement (Table 1). However, the contribution of intestinal P-glycoprotein in drug–drug interactions is generally expected to be minor.^13^

**Table 1:** Overview of the effects of pharmacokinetic interactions involving strong cytochrome P450 (CYP)-enzyme and transporter modulators, acid suppression, and food or fat intake on the maximum concentrations and exposure of small molecule inhibitors.

SMI	Interaction category	Dose	N	Evaluated substance(s)	Mechanism involved	Effect on C_max_	Effect on AUC
Adagrasib^I, II^	CYP-enzymes and transporters	200 mg SD	14	Itraconazole 200 mg QD	CYP3A inhibition	140% ↑	300% ↑
		600 mg SD	12	Rifampicin 600 mg QD	CYP3A induction	88% ↓	95% ↓
	Acid suppression	600 mg SD	–	Pantoprazole (dose -)	Bioavailability	32% ↓	38% ↓
	Food/ fat intake	600 mg SD	–	High fat, high-calorie meal	Bioavailability	20% ↑	38% ↑
Afatinib^II, III^	CYP-enzymes and transporters	40 mg SD	22	Rifampicin 600 mg QD 7D	P-gp induction	22% ↓	34% ↓
		20 mg SD	22	Ritonavir 200 mg BID (1 h before afatinib) 3D	P-gp & BCRP inhibition	38% ↑	48% ↑
		20 mg SD	24	Ritonavir 200 mg BID (simultaneously with afatinib) 3D		4% ↑∗	19% ↑
		40 mg SD	22	Ritonavir 200 mg BID (6 h after afatinib intake) 3D		5% ↑∗	11% ↑
	Acid suppression	30 or 40 mg QD	18	Esomeprazole 40 mg QD (concomitant) 7D	Bioavailability ∼	16% ↓∗	10% ↓∗
		30 or 40 mg QD	18	Esomeprazole 40 mg QD (3 h before afatinib) 7D	Bioavailability ∼	5% ↓∗	1% ↓∗
	Food/ fat intake	40 mg SD	14	High fat/high caloric meal vs. fasted	Bioavailability ↓	50% ↓	39% ↓
Alectinib^II, IV, V^	CYP-enzymes and transporters	600 mg SD	24	Rifampicin 600 mg QD MD	CYP3A induction	51% ↓ Alectinib + M4: 4% ↓	73% ↓ Alectinib + M4: 18% ↓
		300 mg SD	17	Posaconazole 400 mg BID MD	CYP3A inhibition	18% ↑ Alectinib + M4: 7% ↑	75% ↑ Alectinib + M4: 36% ↑
	Acid suppression	600 mg SD	24	Esomeprazole 40 mg QD (30 min after high fat, high calorie meal) MD	Bioavailability ↑	16% ↑ Alectinib + M4: 13% ↑	22% ↑ Alectinib + M4: 17% ↑
	Food/ fat intake	600 mg SD	18	High fat, high calorie meal vs. fasted	Bioavailability ↑	170% ↑ M4: 277% ↑ Alectinib + M4: 231% ↑	192% ↑ M4: 228% ↑ Alectinib + M4: 211% ↑
		600 mg SD	28	High fat meal vs. fasted	Bioavailability ↑	200% ↑ M4: 385% ↑	230% ↑ M4: 250% ↑
		600 mg SD	28	Low fat meal vs. fasted	Bioavailability ↑	50% ↑ M4: 160% ↑	40% ↑ M4: 68% ↑
		600 mg or 450 mg BID (cross-over study)	20	Continental breakfast 7D vs. low fat yoghurt 7D	Bioavailability ↑	–	C_trough-12 h_: 14% ↑
		600 mg or 450 mg BID (cross-over study)	20	Self-chosen lunch 7D vs. low fat yoghurt 7D	Bioavailability ↑	–	C_trough-12 h_: 20% ↑
Aumolertinib^II^	CYP-enzymes and transporters	110 mg SD	32	Rifampicin 600 mg QD MD	CYP3A4 induction	79% ↓ HAS-719: 10% ↓	92% ↓ HAS-719: 73% ↓
		110 mg SD	32	Itraconazole 200 mg BID MD	CYP3A4 & P-gp inhibition	56% ↑ HAS-719: 87% ↓	273% ↑ HAS-719: 69% ↓
	Acid suppression	No clinical studies conducted			Lack of solubility above pH 8.8		
	Food/ fat intake	110 mg SD	20	High fat meal vs. fasted	Bioavailability ↑	∼0%	20% ↑
Binimetinib^II^	CYP-enzymes and transporters	No clinical studies conducted			Partly metabolized by CYP1A2 and CYP2C19	–	–
	Acid suppression	45 mg SD	15	Rabeprazole 20 mg QD 5D	Bioavailability ∼	17% ↓	4% ↑∗
	Food/ fat intake	45 mg SD	12	Low calorie, low fat meal vs. fasted	Bioavailability ∼	29% ↑∗∗	∼0%
		45 mg SD	12	High fat, high calorie meal vs. fasted	Bioavailability ∼	17% ↓∗∗	∼0%
Brigatinib^II, VI^	CYP-enzymes and transporters	180 mg SD	60	Rifampicin 600 mg QD 9D	CYP3A induction	60% ↓	80% ↓
		90 mg SD	60	Itraconazole 200 mg BID 9D	CYP3A inhibition	21% ↑	101% ↑
		90 mg SD	60	Gemfibrozil 600 mg BID 9D	CYP2C8 inhibition	41% ↓	12% ↓
	Acid suppression	No clinical studies conducted			Highly soluble at pH range 1.2–6.8		
	Food/ fat intake	180 mg SD	21	High fat meal vs. fasted	Bioavailability ∼	13% ↓	2% ↓∗
		180 mg SD	8	Fed vs. fasted	Bioavailability ∼	24% ↓	3% ↓∗
Capmatinib^II^	CYP-enzymes and transporters	600 mg SD	27	Itraconazole 200 mg QD 10D	CYP3A inhibition	∼0%	42% ↑
		600 mg SD	26	Rifampicin 600 mg QD 9D	CYP3A induction	56% ↓	67% ↓
	Acid suppression	600 mg SD	20	Rabeprazole 20 mg QD 4D	Bioavailability ↓	38% ↓	25% ↓
	Food/ fat intake	600 mg SD	24	Low fat meal vs. fasted	Bioavailability ↑	11% ↑∗∗	20% ↑∗∗
		600 mg SD	24	High fat meal vs. fasted	Bioavailability ↑	15% ↑∗∗	46% ↑∗∗
Ceritinib^II, VII−IX^	CYP-enzymes and transporters	450 mg SD	19	Ketoconazole 200 mg BID 14D	CYP3A & P-gp inhibition	22% ↑	186% ↑
		750 mg SD	19	Rifampicin 600 mg QD 14D	CYP3A induction & P-gp induction	44% ↓	70% ↓
	Acid suppression	750 mg SD (fasted)	22	Esomeprazole 40 mg QD 6D	Bioavailability ↓	79% ↓	76% ↓
		750 mg SD (fasted)	196 70	PPI usage ≥70% of time	Bioavailability ↓	25% ↓	AUC_0-24 h_: 30% ↓
	Food/ fat intake	500 mg SD	28	Low fat meal vs. fasted	Bioavailability ↑	43% ↑	58% ↑
		500 mg SD	28	High fat meal vs. fasted	Bioavailability ↑	41% ↑	73% ↑
		750 mg SD	24	Low fat low-calorie meal vs. fasted	Bioavailability ↑	42% ↑	39% ↑
		750 mg SD	24	High fat high-calorie meal vs. fasted	Bioavailability ↑	58% ↑	64% ↑
		450 mg (fed), 750 mg (fasted)	67	Low fat meal vs. fasted	Bioavailability ∼	3% ↑∗	AUC_0-24 h_: 4% ↑∗
		600 mg (fed), 750 mg (fasted)	61	Low fat meal vs. fasted	Bioavailability ↑	24% ↑	AUC_0-24 h_: 25% ↑
		750 mg SD	12	Light snack vs. fasted	Bioavailability ↑	45% ↑	54% ↑
Crizotinib^II, X−XII^	CYP-enzymes and transporters	250 mg SD	14	Rifampicin 600 mg QD 14D	CYP3A induction	69% ↓	82% ↓
		150 mg SD	15	Ketoconazole 200 mg BID 16D	CYP3A inhibition	44% ↑	216% ↑
		250 mg BID	15	Dexamethasone (dose -)	CYP3A induction	–	C_trough,ss_: 2% ↓∗
		250 mg QD (steady state)	–	Itraconazole (dose -)	CYP3A inhibition	33% ↑	57% ↑
		250 mg BID	1	Cobicistat 150 mg QD 14D	CYP3A inhibition	–	AUC_0-12 h_ 78% ↑ C_trough,ss_: 164% ↑
	Acid suppression	250 mg SD	16	Esomeprazole 40 mg QD 5D	Bioavailability ↓	2% ↑∗ PF-06260182: 23% ↓	10% ↓∗ PF-06260182: 18% ↓
	Food/ fat intake	250 mg SD	36	High fat meal vs. fasted	Bioavailability ↓	14% ↓	14% ↓
Dabrafenib^II, XIII, XIV^	CYP-enzymes and transporters	75 mg BID 16D	15	Ketoconazole 400 mg QD 4D	CYP3A inhibition	33% ↑ Hydroxy-: 27% ↑ Desmethyl-: 72% ↑ Carboxy-: 21% ↓	AUC0-t: 71% ↑ Hydroxy-: 82% ↑ Desmethyl-: 68% ↑ Carboxy-: 16% ↓
		75 mg BID 16D	16	Gemfibrozil 600 mg QD 4D	CYP2C8 inhibition	2% ↓∗ Hydroxy-: 10% ↓∗ Desmethyl-: 4% ↑∗ Carboxy-: 4% ↑∗	47% ↑ Hydroxy-: 13% ↑∗ Desmethyl-: 3% ↑∗ Carboxy-: 8% ↑∗
		150 mg BID MD	–	Rifampicin 600 mg QD 10D	CYP3A & CYP2C8 induction	27% ↓	34% ↓ Hydroxy-: ∼0% Desmethyl-: 30% ↓ Carboxy-: 73% ↑
	Acid suppression	150 mg BID MD	–	Rabeprazole 40 mg QD MD	Bioavailability ∼	12% ↓	3% ↓
	Food/ fat intake	150 mg SD	14	High fat breakfast	Bioavailability ↓	51% ↓	31% ↓
		150 mg SD	25	Low fat, low calorie breakfast	Bioavailability ↓	14% ↓	14% ↓
Dacomitinib^II, XV^	CYP-enzymes and transporters	45 mg SD	24	Paroxetine 30 mg QD 10D	CYP2D6 inhibition	10% ↑∗	37% ↑
	Acid suppression	45 mg SD	14	Rabeprazole 40 mg QD 7D	Bioavailability ↓	51% ↓	29% ↓
	Food/ fat intake	45 mg SD	24	High fat, high calorie meal	Bioavailability ↑	24% ↑	14% ↑
Encorafenib^II, XVI−XIII^	CYP-enzymes and transporters	50 mg SD	16	Diltiazem 240 mg QD	CYP3A & P-gp inhibition	45% ↑	83% ↑
		50 mg SD	16	Posaconazole 400 mg BID	CYP3A inhibition	68% ↑	183% ↑
		450 mg MD	11	Modafinil 400 mg QD 7D	CYP3A induction	20% ↓	AUC_last_ 24% ↓
	Acid suppression	100 mg SD	15	Rabeprazole 20 mg QD	Bioavailability ∼	6% ↓∗	3% ↓∗
	Food/ fat intake	100 mg SD	40	High fat meal	Bioavailability ∼	36% ↓	4% ↓∗
Ensartinib^I^	CYP-enzymes and transporters	No clinical studies conducted			Ensartinib is extensively metabolized by CYP3A and is a substrate of P-gp	–	–
	Acid suppression	225 mg QD	224	PPI users vs. non PPI users	Bioavailability ∼	3% ↓∗	AUC_ss_ 3% ↓∗
	Food/ fat intake	225 mg QD	–	High fat, high calorie meal	Bioavailability ↓	37% ↓	25% ↓
Entrectinib^II, XIX^	CYP-enzymes and transporters	600 mg SD	20	Rifampicin 600 mg QD 16D	CYP3A induction	56% ↓	77% ↓
		100 mg SD	20	Itraconazole 200 mg QD 10D	CYP3A inhibition	73% ↑	504% ↑
	Acid suppression	600 mg SD	–	Lansoprazole QD 9D (dose 30 mg)	Bioavailability ↓	23% ↓∗∗	25% ↓∗∗
	Food/ fat intake	225 mg SD	45	High fat, high calorie meal	Bioavailability ↑	6% ↑∗	15% ↑
Erlotinib^II, XX−XXVII^	CYP-enzymes and transporters	150 mg SD	12	Rifampicin 600 mg QD 7D	CYP3A induction	29% ↓	66% ↓
		450 mg SD (+rif) vs. 150 mgSD	13	Rifampicin 600 mg QD 7D	CYP3A induction	3% ↑∗	42% ↓
		100 mg SD	–	Ketoconazole (dose -)	CYP3A inhibition	102% ↑	86% ↑
		75 mg QD (+rit) vs. 150 mg SD	9	Ritonavir 200 mg QD	CYP3A inhibition	9% ↓∗ OSI-420: 72% ↓ OSI-413: 52% ↓	AUC_0-24 h_ 1% ↓∗ OSI-420: 69% ↓ OSI-413: 47% ↓
	Acid suppression	Various doses (cross-over study) 7D	18	Esomeprazole 40 mg QD	Bioavailability ↓	56% ↓	AUC_0-24 h_ 47% ↓
		150 mg SD	24	Omeprazole 40 mg QD 7D	Bioavailability ↓	61% ↓	46% ↓
		150 mg SD	24	Ranitidine 300 mg QD 5D (2 h before erlotinib)	Bioavailability ↓	54% ↓	33% ↓
		Various doses (cross-over study) 7D	18	Esomeprazole 40 mg QD	Bioavailability ↓	56% ↓	47% ↓
		150 mg SD	24	Ranitidine 150 mg BID 5D (10 h before and 2 h after erlotinib)	Bioavailability ↓	17% ↓	15% ↓
		150 mg 7D	28	Esomeprazole 40 mg QD + 250 ml cola vs. water	Bioavailability ↑	42% ↑	AUC_0-12 h_ 39% ↑
	Food/ fat intake	150 mg SD	18	High fat, high calorie meal	Bioavailability ↑	64% ↑ OSI-420: 32% ↑	109% ↑ OSI-420: 98% ↑
		Various doses	18	Cow’s milk 250 ml 7D	Bioavailability ∼	6% ↓∗	AUC_0-24 h_ 3% ↓∗
		100 mg QD 7D	22	Fed state	Bioavailability ↓	Day 1: 56% ↑ Day 8: 33% ↑∗	Day 1: 66% ↑ Day 8: 34% ↑∗
		150 mg 7D	28	250 ml cola vs. water	Bioavailability ∼	0%	AUC_0-12 h_ 9% ↑
		150 mg 7D	23	Breakfast	Bioavailability ↑	44% ↑	33% ↑
Gefitinib^II, XX^^V^^III−XXX^	CYP-enzymes and transporters	500 mg SD	18	Rifampicin 600 mg 16D	CYP3A induction	65% ↓	83% ↓
		250 mg SD	18	Phenytoin 5 mg/kg/day 5D	CYP3A induction	26% ↓	47% ↓
		500 mg SD	48	Itraconazole 200 mg QD 12D	CYP3A inhibition	32% ↑	61% ↑
		250 mg SD	48	Itraconazole 200 mg QD 12D	CYP3A inhibition	51% ↑	78% ↑
	Acid suppression	250 mg SD	26	Ranitidine 450 mg BID	Bioavailability ↓	71% ↓	AUC_0-24 h_ 47% ↓
		250 mg MD	47	PPI (15 or 30 mg lansoprazole, 20 mg omeprazole or 20 mg esomeprazole)	Bioavailability ↓	35% ↓	AUC_0-24 h_ 35% ↓
		250 mg MD	47	20 or 40 mg famotidine or 300 mg nizatidine	Bioavailability ↓	20% ↓∗	AUC_0-24 h_ 21% ↓∗
	Food/ fat intake	250 mg SD	26	High fat breakfast	Bioavailability ↑	32% ↑	37% ↑
Larotrectinib^II^	CYP-enzymes and transporters	100 mg SD	12	Rifampicin 600 mg SD	P-gp & BCRP inhibition	80% ↑	70% ↑
		100 mg SD	12	Rifampicin 600 mg BID 11D	CYP3A induction	71% ↓	81% ↓
		100 mg SD	12	Itraconazole 200 mg QD 8D	CYP3A inhibition	180% ↑	330% ↑
	Acid suppression	No clinical studies conducted			Fully soluble at pH range 1.2–6.8	–	–
	Food/ fat intake	100 mg SD	18	High fat, high calorie meal	Bioavailability ↓	34% ↓	∼0%
Lazertinib (+amivantamab)^II^	CYP-enzymes and transporters	240 mg SD	32	Rifampicin 600 mg QD 17D	CYP3A induction	72% ↓	AUC_0-120 h_ 83%↓
		160 mg SD	32	Itraconazole 200 mg QD 7D	CYP3A inhibition	19% ↑	AUC_0-120 h_ 46%↑
	Acid suppression	240 mg MD	33	Acid reducing agents ≥4D	Bioavailability ∼	10% ↓∗	AUCss 9% ↓∗
	Food/ fat intake	240 mg SD	24	High fat meal	Bioavailability ∼	7% ↓∗	AUC_last_ 14% ↑∗
Lorlatinib^II, XXXI, XXXII^	CYP-enzymes and transporters	100 mg SD	12	Rifampicin 600 mg QD 12D	CYP3A induction	76% ↓	85% ↓
		100 mg SD	16	Itraconazole 200 mg QD 11D	CYP3A inhibition	24% ↑	42% ↑
		100 mg SD	16	Modafinil 400 mg QD 19D	CYP3A induction	22% ↓	23% ↓
	Acid suppression	100 mg SD	27	Rabeprazole 20 mg QD 5D	Bioavailability ∼	30% ↓	1% ↑∗
	Food/ fat intake	100 mg SD	27	High fat, high calorie meal	Bioavailability ∼	9% ↓	5% ↑
Osimertinib^II, XXXIII, XXXIV^	CYP-enzymes and transporters	80 mg MD	–	Rifampicin 600 mg QD 21D	CYP3A induction	73% ↓ AZ5104: 78% ↓	78% ↓ AZ5104: 82% ↓
		80 mg SD	–	Itraconazole 200 mg BID 13D	CYP3A inhibition	20% ↓ AZ5104: 24% ↓	24% ↑ AZ5104: 8% ↑
		80 mg QD 160 mg QD	10 1	Cobicistat 150 mg QD	CYP3A inhibition	–	AUC_0-24 h_, ss osimertinib + _AZ5104_ 60%↑
	Acid suppression	80 mg SD	68	Omeprazole 40 mg QD 5D	Bioavailability ∼	2% ↑∗	7% ↑
	Food/ fat intake	80 mg SD	38	Fed state	Bioavailability ∼	7% ↓∗	6% ↑∗
Pralsetinib^II, XXXV^	CYP-enzymes and transporters	400 mg SD	25	Rifampicin 600 mg QD 16D	CYP3A & P-gp induction	30% ↓	68% ↓
		200 mg SD	25	Itraconazole 200 mg QD 14D	CYP3A & P-gp inhibition	84% ↑	250% ↑
		200 mg SD	15	Cyclosporine 600 mg SD	P-gp inhibition	48% ↑	81% ↑
	Acid suppression	400 mg SD	36	Esomeprazole 40 mg QD 6D	Bioavailability ↓	25% ↓	15% ↓
	Food/ fat intake	200 mg SD	20	High fat, high calorie meal	Bioavailability ↑	104% ↑	122% ↑
Repotrectinib^II^	CYP-enzymes and transporters	160 mg SD	14	Rifampicin 600 mg QD	CYP3A & P-gp induction	80% ↓	>90% ↓
		80 mg SD	16	Itraconazole 200 mg QD	CYP3A & P-gp inhibition	167% ↑	589% ↑
	Acid suppression	No clinical studies conducted			pH independent solubility across pH range 1.2–7.4	–	–
	Food/ fat intake	160 mg SD	28	High fat, high calorie meal (vs. modified fasted; no food and drinks 1 h before and 2 h after intake)	Bioavailability ↑	43% ↑	34% ↑
		160 mg SD	15	High fat meal	Bioavailability ↑	110% ↑	42% ↑
		160 mg SD	13	Low fat meal	Bioavailability ↑	124% ↑	36% ↑
Selpercatinib^I, II^	CYP-enzymes and transporters	160 mg SD	–	Rifampicin 600 mg SD	P-gp inhibition	19% ↑	6% ↑
		160 mg SD	–	Rifampicin 600 mg QD 11D	CYP3A induction	70% ↓	87% ↓
		160 mg SD	–	Itraconazole 200 mg QD 7D	CYP3A inhibition	30% ↑	130% ↑
	Acid suppression	160 mg SD	20	Omeprazole 40 mg QD (fasted)	Bioavailability ↑	88% ↓	69% ↓
		160 mg SD	20	Omeprazole 40 mg (fed)	Bioavailability ∼	22–50% ↓	∼0%
		160 mg SD	–	Ranitidine 10 h prior to and 2 h after selpercatinib dose	Bioavailability ∼	18% ↓	7% ↓
	Food/ fat intake	160 mg SD	20	High fat breakfast	Bioavailability ∼	14% ↓∗	9% ↑∗
Sevabertinib^I^	CYP-enzymes and transporters	10 mg SD	–	Itraconazole 200 mg QD MD	CYP3A inhibition	60% ↑	130% ↑
		40 mg SD	–	Carbamazepine 600 mg QD MD	CYP3A induction	57% ↓	79% ↓
	Acid suppression	20 mg SD	–	Esomeprazole 40 mg QD 5D	Bioavailability ∼	29% ↓∗	10% ↓∗
	Food/ fat intake	20 mg SD	–	High fat meal	Bioavailability ↓	56% ↓	28% ↓
		20 mg SD	–	Low fat meal	Bioavailability ↓	28% ↓	16% ↓
Sotorasib^II, XXXVI−XXXVIII^	CYP-enzymes and transporters	960 mg SD	14	Rifampicin 600 mg QD 15D	CYP3A induction	35% ↓	51% ↓
		360 mg SD	14	Itraconazole 200 mg 5D	CYP3A inhibition	4% ↑∗	26% ↑
	Acid suppression	960 mg SD	14	Omeprazole 40 mg QD 6D (fasted)	Bioavailability ↓	57% ↓	42% ↓
		960 mg SD	14	Omeprazole 40 mg QD 6D (fed state, standard calorie, moderate-fat meal)	Bioavailability ↓	65% ↓	57% ↓
		960 mg SD	14	Omeprazole 40 mg QD 6D (with acidic beverage – cola)	Bioavailability ↓	32% ↓	23% ↓
		960 mg SD	14	Famotidine 40 mg (10 h prior to and 2 h after sotorasib, fed state)	Bioavailability ↓	35% ↓	38% ↓
	Food/ fat intake	360 mg SD	14	High fat meal	Bioavailability ↑	3% ↑∗	38% ↑
		960 mg SD	8	High fat meal	Bioavailability ↑	34% ↓	25% ↑
		360 mg MD	2	Fed state	Bioavailability ↑	38% ↑	75% ↑
Sunvozertinib^I^	CYP-enzymes and transporters	100 mg SD	19	Itraconazole 200 mg QD (low fat fed state)	CYP3A & P-gp inhibition	32% ↑	51% ↑
		300 mg SD	20	Carbamazepine (dose -)	CYP3A & P-gp induction	38% ↓	48% ↓
	Acid suppression	–	–	–	pH dependent solubility	Population PK analysis showed that PPI use does not significantly affect drug absorption.	
	Food/ fat intake	300 mg SD	20	High fat meal	Bioavailability ∼	11% ↑	9% ↑
Taletrectinib^I, XXXIX^	CYP-enzymes and transporters	200 mg SD	28	Itraconazole 200 mg QD 11D	CYP3A & P-gp inhibition	80% ↑	230% ↑
		200 mg SD	28	Rifampicin 600 mg QD 19D	CYP3A & P-gp induction	42% ↓	86% ↓
	Acid suppression	400 mg SD	22	Omeprazole 40 mg QD 5D	Bioavailability ↓	65% ↓	40% ↓
	Food/ fat intake	400 mg SD	32	High fat meal	Bioavailability ↑	50% ↑	50% ↑
		400 mg SD	11	Low fat meal	Bioavailability ↑	45% ↑	AUC_0–24h_ 23% ↑
Tepotinib^II, XL^	CYP-enzymes and transporters	500 mg SD	16	Itraconazole 200 mg QD 11D	CYP3A & P-gp inhibition	2% ↑∗	22% ↑
		500 mg SD	14	Carbamazepine 300 mg BID 21D	CYP3A & P-gp induction	11% ↓	35% ↓
	Acid suppression	500 mg SD	12	Omeprazole 40 mg QD 5D	Bioavailability ↑	4% ↑∗	10% ↑
	Food/ fat intake	500 mg SD	12	High fat meal	Bioavailability ↑	100% ↑	60% ↑
Trametinib^II, XIV^	CYP-enzymes and transporters	No clinical studies conducted			Mainly metabolized via deacetylation, mono-oxygenation and glucuronidation. CYP3A is considered a minor metabolic pathway.	–	–
	Acid suppression	No clinical studies conducted			Low solubility across physiological pH range	–	–
	Food/ fat intake	2 mg SD	24	High fat meal	Bioavailability ↓	70% ↓	10% ↓
		2 mg SD	24	Low fat, low calorie breakfast	Bioavailability ↓	44% ↓	18% ↓
		2 mg MD (steady state)	–	Fed state (predicted data)	Bioavailability ↓	46% ↓	24% ↓
Zongertinib^I, XLI^	CYP-enzymes and transporters	60 mg SD	15	Carbamazepine 600 mg QD 14D	CYP3A & P-gp induction	43% ↓	63% ↓
		15 mg SD	16	Itraconazole 200 mg QD 14D	CYP3A & P-gp inhibition	27% ↑	41% ↑
	Acid suppression	30 mg SD	12	Rabeprazole 40 mg 5D	Bioavailability ∼	13% ↓∗	AUC_last_ 3% ↓∗
	Food/ fat intake	240 mg SD	16	High fat, high calorie meal	Bioavailability ↑	26% ↑	26% ↑

Auto-inhibitors of CYP-enzymes: adagrasib, ceritinib, crizotinib, larotrectinib, lazertinib (weak), pralsetinib, selpercatinib (weak), sevabertinib and taletrectinib; Auto-inducers of CYP-enzymes: brigatinib (weak), dabrafenib, encorafenib, lorlatinib, osimertinib, repotrectinib, sotorasib (weak) and sunvozertinib; In the case of multiple doses, full dosing periods were reported if available; Abbreviations: Roman numerals, corresponding references are provided in the Supplementary; SMI, small molecule inhibitor; PK, pharmacokinetic; CYP, cytochrome P450; C_max_, maximum plasma concentration; AUC, area under the curve; AUC_last_, area under the curve from time zero to the last quantifiable time point; AUC_0-h_, area under the curve from time zero to the mentioned timepoint; C_trough_, trough concentration; ss, steady state; QD, once daily; BID, twice daily; SD, single dose; MD, multiple dose; h, hour(s); D, day(s); vs, versus; rif, rifampicin; rit, ritonavir; PPI, proton pump inhibitor; BCRP, breast cancer resistance protein; P-gp, P-glycoprotein; M4, metabolite of alectinib; HAS-719, metabolite of aumolertinib; PF-06260182, metabolite of crizotinib; hydroxyl-, desmethyl-, carboxy-dabrafenib, metabolites of dabrafenib; OSI-420 and OSI-413, metabolites of erlotinib; AZ5104, metabolite of osimertinib; ↓ reduced; ↑ increased; ∼ similar; – unknown or not studied; ∼0% no relevant effect observed; ∗ not significant; ∗∗ significance unknown.

Variations in systemic drug exposure become clinically significant when they deviate from the FDA and EMA bioequivalence range of 80–125%.^16^^,^^17^ The efficacy of pharmacological agents generally depends on maintaining plasma concentrations within a defined therapeutic window. Subtherapeutic drug levels may lead to reduced clinical efficacy, while exceeding the upper limit of this range is typically associated with an increased risk of toxicity.^18^ Table 1 provides an overview of studies investigating the pharmacokinetic interactions involving strong modulators of CYP-enzymes, transporters, acid-reducing agents and food, and their effect on the pharmacokinetics of SMIs approved for NSCLC. Notably, most drug-interaction studies were conducted with single doses of SMIs. For auto-inducing or auto-inhibiting SMIs, these results may not reflect steady-state conditions, as clearance can change with repeated dosing. Fig. 1 shows an overview of clinically relevant pharmacokinetic interactions affecting SMIs used in the treatment of NSCLC. Table 2 presents clinical practice recommendations for each SMI, based on the data summarised in Table 1 and the corresponding FDA/EMA labels. Most drug–drug interaction studies have been conducted using potent CYP-enzyme inducers or inhibitors, such as rifampicin and itraconazole. Therefore, recommendations primarily emphasise strong CYP-enzyme modulators, from which guidance for less potent modulators can be derived.

**Table 2:** Clinical implications of pharmacokinetic (PK) and pharmacodynamic (PD) interactions with small molecule inhibitors (SMIs).

Cell colours reflects the severity of the interaction as assigned by expert opinion (green, safe (PK interaction) or relatively low incidence (PD interaction); orange = caution (PK interaction) or relatively moderate incidence (PD interaction); red, contra-indicated (PK interaction) or relatively high incidence (PD interaction)); [number(s)] indicate the supporting evidence categories: 1. High-level evidence (pharmacokinetic studies, systematic reviews, meta-analyses, and pooled analyses of prospective clinical trials, providing direct support for the recommendation), 2. Moderate-level evidence (phase I/II clinical trials, non-randomized prospective studies, observational studies, real-world studies, retrospective cohort studies, and post-hoc analyses), 3. Regulatory documents (FDA and/or EMA labels, product information, assessment reports and review files), 4. Expert-based evidence (expert consensus statements, position papers, narrative reviews, and other publications in which recommendations are primarily based on expert opinion and interpretation of the available evidence rather than direct clinical evidence), 5. Authors’ expert opinion (formulated by the authors based on review and interpretation of the available evidence when no direct supporting evidence or external expert guidance is available); other PD interactions include weight gain, keratitis, uveitis, retinal pigment epithelial detachment, conjunctivitis, non-infectious pyrexia, stomatitis, colitis and neurological toxicity; SMI, small molecule inhibitor; ALK, anaplastic lymphoma kinase; BRAF, B-Raf proto-oncogene; EGFR, epidermal growth factor receptor; KRAS (G12C), K-Ras proto-oncogene; MEK, mitogen-activated protein kinase; MET, mesenchymal–epithelial transition; NTRK, neurotrophic tropomyosin receptor kinase; RET rearranged during transfection; ROS1, ROS proto-oncogen 1 tyrosine kinase receptor; ∗ = ROS1 and NTRK; HER2, human epidermal growth factor receptor 2; PK, pharmacokinetic; PD, pharmacodynamic; QD, once daily; CYP, cytochrome P450-enzyme; P-gp, P-glycoprotein; BCRP, breast cancer resistance protein; OATP1B1/3, organic anion transporting polypeptide 1B1 and 1B3; ASA, acid suppressive agent; PPI = proton pump inhibitor; H2-antagonist, histamine 2 receptor antagonist; GLP1, glucagon-like peptide-1; NSAID, nonsteroidal anti-inflammatory drug; GI-toxicity, gastrointestinal toxicity; ECG, electrocardiogram; QTc, corrected QT interval; TdP, torsades de pointes; VT, ventricular tachycardia; ILD, interstitial lung disease; RPED, retinal pigment epithelial detachment; SPC, summary of product characteristics.

### Food-drug interactions

Food–drug interactions are a means to improve the safety and pharmacokinetics of SMIs in patients with NSCLC. For example, ceritinib was initially approved at a dose of 750 mg once daily under fasted conditions. Subsequent studies demonstrated that administering 450 mg once daily with food resulted in comparable systemic exposure while significantly reducing gastrointestinal toxicity.^14^^,^^19^ Similarly, co-administration of adagrasib and taletrectinib with food significantly mitigates gastrointestinal toxicity.^14^^,^^15^ As taletrectinib is currently approved for administration in a fasted state, the FDA has required a post-marketing evaluation of dosing with food.^15^

A few SMIs that are registered for administration in fasted state exhibit increased systemic exposure when administered with food, indicating potential for optimisation in cases of subtherapeutic exposure. Erlotinib, for example, demonstrates a 34–109% increase in exposure when taken with food.^14^^,^^20^^,^^21^ Additionally, certain SMIs currently approved without food restrictions, including adagrasib, aumolertinib, capmatinib, gefitinib, repotrectinib, and sotorasib, show an increase in area under the curve of ≥20% when taken with food.^14^ Food intake also reduces the inter-individual variability in exposure of adagrasib, binimetinib, capmatinib, erlotinib and zongertinib, which enhances the predictability of therapeutic outcomes by increasing the proportion of patients achieving therapeutic exposure levels.^14^^,^^20^^,^^22^

For some SMIs for which pharmacokinetics are influenced by food, the type of meal (*e.g.,* low-fat or high-fat) may significantly affect systemic exposure.^14^ For erlotinib, a high-fat/high-calorie meal substantially increased exposure, whereas 3% fat cow’s milk had no clinically relevant effect.^20^^,^^23^ This distinction is particularly notable for the poorly aqueous-soluble alectinib. Co-administration with a low-fat meal resulted in a 40% increase in systemic exposure compared to fasting, whereas a high-fat meal resulted in a 240% increase, indicating enhanced dissolution and intestinal permeability with higher-fat foods.^23^ This was confirmed in another study, in which 35% of patients failed to reach the alectinib exposure threshold when administered with low-fat yoghurt, compared to only 5% of patients receiving a continental breakfast or lunch.^24^ Thus, even for SMIs registered to be administered with food, adjusting meal composition should be considered upon subtherapeutic exposure. However, certain SMIs (*e.g.,* alectinib) are associated with weight gain, which complicates the use of high-fat meals to enhance drug exposure.^14^^,^^15^^,^^25^

### Acid suppressive agents

Several SMIs exhibit reduced systemic exposure when co-administered with acid-suppressive agents such as proton pump inhibitors (PPIs). This observation prompts investigation into whether concurrent food intake, particularly for SMIs for which absorption is enhanced by food, may partially mitigate this reduction in exposure. However, a limited number of studies have explored the combined effect of food and acid suppression on the exposure of SMIs used in NSCLC treatment. Selpercatinib demonstrates a notable benefit from food intake under acid-suppressive conditions; co-administration with both a PPI and food maintains systemic exposure, whereas co-administration of a PPI under fasted conditions results in a ∼70% reduction in exposure.^14^ In contrast, sotorasib shows an exacerbated decrease in systemic exposure when administered with both food and a PPI, exhibiting a 57% reduction compared to a 42% decrease with PPI use in fasted state.^26^ The reduction in drug exposure observed with PPI use under fasted conditions was attenuated to 23% when water was substituted with an acidic beverage, by temporarily decreasing gastric pH.^26^ A similar pattern has been observed with erlotinib combined with a PPI, where the highly acidic beverage cola resulted in a ∼40% increase in drug exposure, largely mitigating the negative effect of the PPI on erlotinib exposure.^27^

Concurrent ingestion of food or acidic beverages may attenuate the PPI-induced reduction in systemic exposure of SMIs; however, this effect is drug-specific and not universally observed. When PPI use is unavoidable, the optimal strategy should be guided by the SMIs pharmacokinetics, weighing options such as co-administration with food, concurrent intake with an acidic beverage and staggered dosing.

## Pharmacodynamic effects

**Fig. 2:**
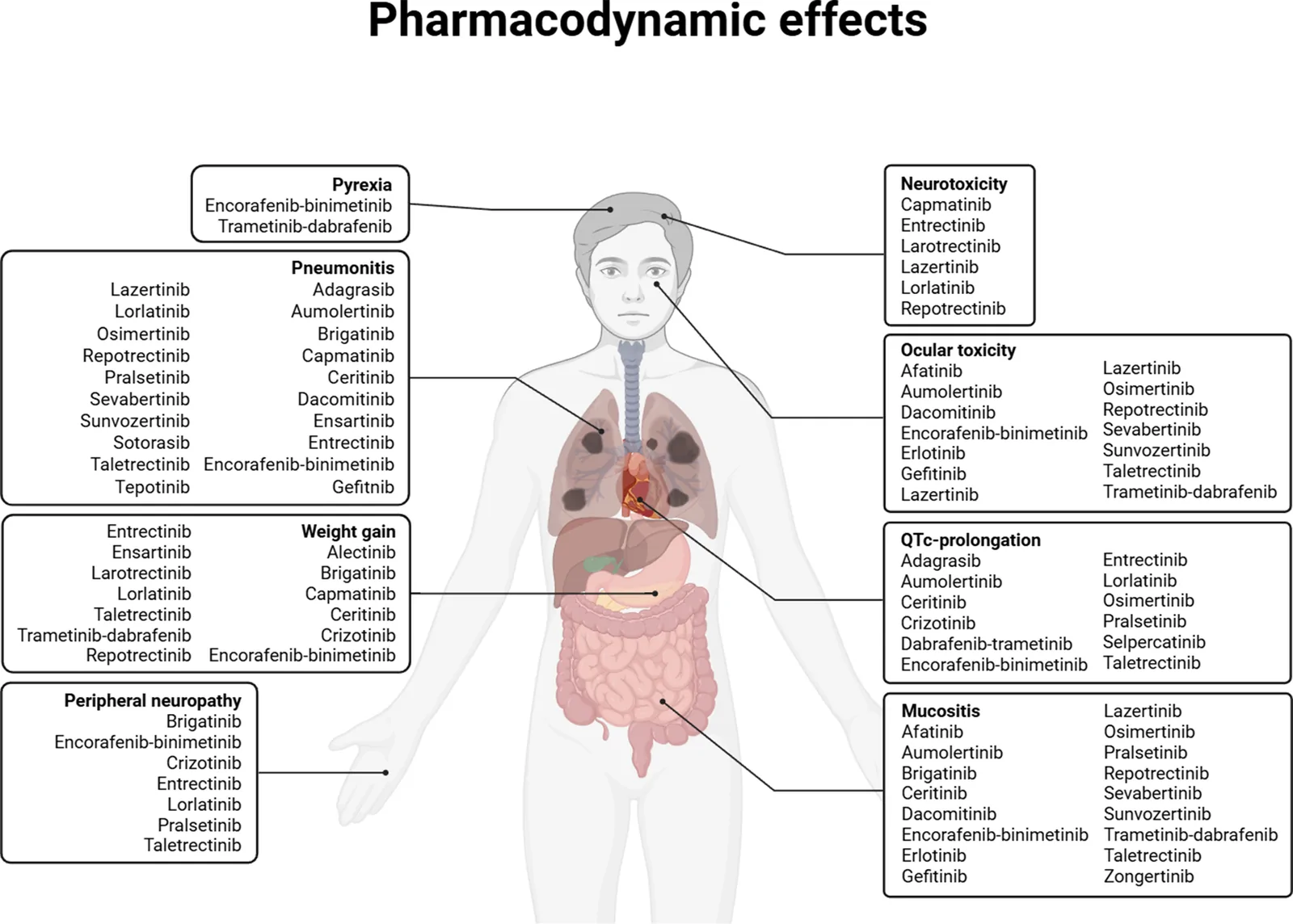
**A comparative overview of clinically relevant pharmacodynamic effects associated with small molecule inhibitors used in the treatment of non-small cell lung cancer.** Effects include corrected QT interval (QTc)-prolongation, pneumonitis, weight gain, ocular toxicity, neurotoxicity, peripheral neuropathy, pyrexia, colitis and mucositis.

Unwanted pharmacodynamic effects causing toxicity of SMIs are complex and pose additional clinical challenges.^5^ Toxicity can be broadly categorised into on-target effects, which depend on the distribution of the targeted pathways across various tissues (*e.g.,* rash from epidermal growth factor receptor (EGFR) SMIs), and off-target effects due to the unintended inhibition of kinases other than the target.^5^ Also, physicochemical properties, including ionisation potential, lipophilicity, and plasma protein binding contribute to the volume of distribution, which may facilitate tissue accumulation.^28^ Thus, toxicity profiles differ not only between distinct SMI classes but also among SMIs within their class. Although the adverse effects of SMIs have been studied in general, comparative evaluations of clinically relevant toxicities, including QTc-prolongation, pneumonitis, ocular toxicity (keratitis and conjunctivitis), weight gain, neurological toxicity, pyrexia, colitis and stomatitis, and hepatotoxicity, and clinical management strategies remain underexplored.^5^^,^^29^ Fig. 2 shows an overview of clinically relevant pharmacodynamic effects of SMIs used NSCLC treatment. A detailed overview of the incidence, clinical presentation and clinical relevance of QTc-prolongation, pneumonitis, and ocular toxicity (keratitis and conjunctivitis) is presented in Table 3. The reported adverse event frequencies in this manuscript were predominantly derived from separate studies and regulatory documents rather than head-to-head trials and should therefore be interpreted with caution. In Table 2 we present practical considerations for pharmacodynamic effects.

**Table 3:** Overview of the incidence (Common Terminology Criteria for Adverse Events (CTCAE) all grades and grade ≥3) of corrected QT interval (QTc) prolongation, pneumonitis, and ocular toxicity associated with small molecule inhibitors (SMIs) in the treatment of non-small cell lung cancer (NSCLC).

Target	SMI	QTc-prolongation							Pneumonitis			Ocular toxicity			
		N	Incidence (%)					ΔQTc (ms)	N	Incidence (%)		Type	N	Incidence (%)	
			All gr	Gr ≥ 3	Δ > 30–60 ms	Δ > 60 ms	QTc >500 ms			All gr	Gr ≥ 3			All gr	Gr ≥ 3
ALK	Alectinib^I, II, XLII^	221	–	–	12.8	0.5	0.5	+5.3 ms at C_max,ss_ (one-sided upper 95% CI: +7.2 ms)	253	0.4	0.4	No known records			
		7∗	–	–	–	–	–	+12.1 ms (90% CI: 4.5, 19.8 ms)	128	3.1	0.8				
		53	0.1	0.0	–	–	–		533	1.3	0.4				
	Brigatinib^I, II, XLIII^	219	6.0	0.7	–	–	–	<10.0 ms −0.1 ms (range −5.1, +8.8 ms)	219	9.0	4.0	No known records			
		123∗	–	–	–	–	–		136	3.7	–				
		–∗	–	–	–	–	–								
		–∗	–	–	15.0	0.0	0.0		51	11.8	6.0				
	Ceritinib^I, II^	150	–	–	–	–	–	+17.0 ms at C_max_ (90% CI: 14.9, 19.1 ms) +21.2 ms at 75th percentile of C_max_ (90% CI: 18.8, 23.5 ms)	925	2.1	1.2	No known records			
		925	9.7	–	–	–	–		525	3.4	1.9				
		525	6.1	0.8	–	4.4	0.2								
		955	–	–	–	–	–	+18.2 ms (90% CI: 17.2, 19.2 ms)							
	Crizotinib^I, II, XLIV^	386	1.0	0.5	–	4.1	1.0	+12.3 ms at cycle 2 (90% CI: 5.1, 19.5 ms) +7.5 ms at mean C_max_ (90% CI: 2.3, 12.8 ms)	386	1.0	1.0	No known records			
		52	–	–	21.0	2.0	0.0								
								
		62	–	–	–	–	–		2028	5.8	3.5				
		1619∗	–	–	–	5.0	2.1		1669∗	1.2	–				
		1722∗	4.0	1.6	–	–	–								
	Ensartinib^I^	141	–	–	2.1	0.7	0.0	−4.9 ms at C_max_ (90% CI: −6.4, −3.4 ms)	458	5.0	1.7	No known records			
		458	0.9	0.0	–	–	–								
									
									
	Lorlatinib^I, II^	295	5.4	–	–	–	–	−21.4 ms at Cmax,ss −25.9 ms at Cmax,SD	295	1.4	1.0	No known records			
							547	2.4	0.7						
		327	–	–	–	1.8	0.7								
		275	–	–	–	–	–	+10.1 ms (90% upper CI 12.1 ms)							
BRAF (+MEK)	Dabrafenib^II, XLV^	31	–	–	3.0	0.0	0.0	+2.9 ms at C_max_ (90% CI: 1.4, 7.1 ms)	No known records			Uveitis	578	0.7	–
		–∗	–	–	16.0	3.0	0.2					Iritis	578	0.2	–
	Encorafenib^I, II^	19	–	–	–	–	–	+19.9 ms at day 15 (90% CI 13.3, 26.5 ms) Max. +33.4 ms (upper 90% CI 38.8 ms)	No known records			Uveitis	217∗	0.5	0.0
		105∗	–	–	40.0	4.8	0.0								
		204∗	–	–	–	3.4	2.5								
	Binimetinib^I, II^	44∗	–	–	–	–	–	+4.7 ms (90% CI: −1.1, 10.5 ms)	–∗	1.4	–	No known records			
		94∗	–	–	8.6	2.2	1.0								
	Trametinib^I^	50	–	–	–	–	–	+3.2 ms at C_max_ (90% CI: 0.3, 6.0 ms)	No known records			No known records			
	Dabrafenib + trametinib^II, XLVI^	55	–	–	13	2.0	0.0		82	1.2	0.0	Uveitis	641	0.9	–
												Uveitis	551∗	0.5	–
	Encorafenib + binimetinib^I, II, XLVI^	66	–	–	–	–	–	+18.3 ms at day 15 (90% CI 14.4, 22.1 ms)	98	2.0	0.0	Uveitis	192	3.6	0.5
		98	5.1	–	–	7.3	2.1		192	NA	0.5	Uveitis	372	3.5	0.3
		268	–	–	–	4.9	0.7		257	NA	0.4	Uveitis	257∗	3.9	NA
		372	–	–	–	6.0	1.1					RPED	372	22.3	1.6
												RPED	274	29.6	1.8
EGFR	Afatinib^II, XLVII^	60	–	–	–	–	–	−0.3 ms at day 14 (90% CI: −2.8, 2.3 ms)	3865∗	0.7	0.5	Keratitis	1841∗	1.3	–
									1602∗	4.4	1.6	Keratitis	353∗	2.8	–
	Aumolertinib^II^	545	9.4	0.7	–	4.2	0.9	+9.1 ms (90% CI: 7.1, 11.0 ms)	545	2.9	0.2	Conjunctivitis	545	3.1	0.0
		244	–	–	30.3	3.3	–								
		23	–	–	–	–	–								
	Dacomitinib^I, II^	32	–	–	–	–	–	+5.2 ms (90% CI: 1.2, 9.2 ms)	227	2.7	0.8	Keratitis	227	1.8	0.4
		227	0.4	–	–	–	–					Conjunctivitis	227	24.2	0.0
		–∗	–	–	–	2.8	0.2								
	Erlotinib^II, XLVIII, XLIX^	21∗	–	–	–	–	–	+9 ms (range 7, 24 ms) No effect on QTc at any dose	485∗	0.8	–	Conjunctivitis	485	12.0	0.6
												Keratitis	473	3.2	–
		12∗	–	–	–	–	–								
	Gefitinib^I, II, XLIX, LI^	246∗	–	–	–	–	–	No clinically significant effect on QTc	607	2.6	1.4	Keratitis	570∗	1.6	–
		160	–	–	–	–	–	−6.5 ms (90% CI: −20.8, 7.8 ms)	5368	3.6	–	Conjunctivitis	729	2.9	–
		10	–	–	–	–	–	+6.0 ms (p > 0.05)	336	15.2	–				
		85	–	–	6.2	0.0	0.0								
	Lazertinib (+amivantamab)^I, II^	–	–	–	–	–	–	+3.5 ms (90% CI: 1.1, 5.9 ms)	421	3.1	1.4	Keratitis	421	2.6	0.5
									550	2.4	1.3				
		421	3.3	–	–	0.0	0.3								
	Osimertinib^I, II, XLVIII, LII−LVI^	210	3.3	–	35.7	2.9	0.5	+16.2 ms (90% CI: 14.8, 17.6 ms) +14.0 ms (upper 90% CI 16.0 ms) (PK/PD modelling) +0.3 ms per 10 nM increase in plasma concentration	411	2.7	1.0	Conjunctivitis	411	2.9	–
		411	4.1	1.2	–	2.7	0.2		3015	0.9	0.4	Keratitis	5253	0.6	–
		3015	2.7	0.0	–	–	–		1221∗	2.9	–	Keratitis	153	2.6	–
		116∗	7.9	–	–	–	–		538∗	16.7	5.2				
		538∗	4.6	1.3	–	–	–		1700	1.1	–				
		1700	2.2	–	–	–	–								
	Sunvozertinib^I^	311∗	5.8	1.0	18.5	2.0	1.5	+0.9 ms at C_max_ (90% CI: −0.5, 2.3 ms)	121	1.7	0.8	Keratitis	121	0.8	–
									311∗	5.5	3.6		311∗	1.0	0.3
HER2	Sevabertinib^I^	–	–	–	–	–	–	+1.5 ms at C_max,ss_ (upper 90% CI 2.6 ms)	268	0.7	0.4	Conjunctivitis	268	3.4	0.0
		268	2.2	0.7	–	–	–					Keratitis	268	0.4	0.0
	Zongertinib^I^	–	–	–	–	–	–	+0.5 ms at C_max_ (90% CI: −0.2, 1.2 ms)	448∗	0.8	0.0	No known records			
		448∗	1.6	0.0	–	–	–								
		423∗	–	–	9.0	0.5	0.0								
KRAS	Adagrasib^II^	260	15.4	5.0	46.3	13.2	6.6	+17.9 ms at C_max,ss_ (90% CI: 15.1, 20.7 ms)	260	5.4	1.9	No known records			
									366	4.1	1.4				
	Sotorasib^I, II, LVII, LVIII^	200	–	–	9.0	0.0	0.0		740	1.9	0.8	No known records			
		–	–	–	–	–	–	+8.1 ms (90% CI: 5.8, 10.5 ms)	105	NA	1.9				
									549	1.6	NA				
MET	Capmatinib^I, II^	408	2.7	0.8	–	0.3	0.0	+2.3 ms (at the 75th percentile of Cmax) +1.3 ms at mean steady state C_max_ (90% CI: 0.1, 2.6 ms)	373	5.4	2.1	No known records			
									160	6.9	4.4				
	Tepotinib^I, II^	285	–	–	–	–	–	+2.0 ms (90% CI: 0.5, 3.6 ms)				No known records			
		291	2.1	–	–	5.2	2.1		291∗	2.7	–				
		–	–	–	–	–	–	+3.3 ms (90% CI: 1.2, 5.4 ms)							
NTRK & ROS1	Entrectinib^I, II, LIX^	80	–	–	–	–	–	−3.9 ms at cycle 3 (90% CI: −8.2, 0.4 ms)	504∗	2.0	0.8	No known records			
		853∗	3.6	1.9		7.2	4.1		269∗	1.9	0.7				
		355∗	–	–	–	2.8	1.7								
		269∗	1.5	0.4	–	–	–								
	Larotrectinib^1,I, II^	–	–	–	–	–	–	−13.2 ms (range 10.0–15.6 ms) for 200 mg SD, 100 mg BID (PK/PD modelling) −3.3 ms (upper 90% CI −2.1 ms)	No known records			No known records			
		48	–	–	0.0	0.0	0.0								
	Repotrectinib^I, II^	367∗	1.1	–	19.1	1.6	0.3	+8.3 ms (90% CI: 6.6, 10.0 ms)	367∗	3.5	–	Conjunctivitis	367∗	0.8	–
		565∗	0.9	–	19.3	1.1	0.4		565∗	3.2	0.9				
	Taletrectinib^I^	351	21.0	5.1	–	13.0	2.6	+12.8 ms in fasted state (90% CI upper bound 15.4 ms) +20.5 ms in fed state (90% CI 16.3, 24.7 ms)	337	2.4	1.2	Conjunctivitis	352	1.4	–
		41	–	–	–	–	–								
RET	Selpercatinib^I, II^	746∗	18.1	4.0	–	–	–		No known records			No known records			
		679∗	–	–	–	15.0	5.6								
		–	–	–	–	–	–	+10.6 ms (90% CI: 9.1, 12.1 ms)							
	Pralsetinib^I, II^	528	5.1	0.4	–	–	–		528	11.6	3.0	No known records			
		34	–	–	–	–	–	+5.6 ms mean change at Css (range 4.9–7.7 ms)							
		34	–	–	–	0.0	0.0	+4.3 ms (90% CI: −1.4, 9.9 ms)							

For QTc-prolongation, the change from baseline (ΔQTc) in milliseconds (ms) observed in registration studies is presented.

Pneumonitis and interstitial lung disease (ILD) are being used interchangeably. Abbreviations: Roman numerals, corresponding references are provided in the Supplementary. SMI, small molecule inhibitor; ALK, anaplastic lymphoma kinase; BRAF, B-Raf proto-oncogene; EGFR, epidermal growth factor receptor; KRAS (G12C), K-Ras proto-oncogene; HER2, human epidermal growth factor receptor 2; MEK, mitogen-activated protein kinase; MET, mesenchymal–epithelial transition; NTRK, neurotrophic tropomyosin receptor kinase; ROS1, ROS proto-oncogen 1 tyrosine kinase receptor; ^1^ = selective NTRK inhibitor; RET, rearranged during transfection; PK, pharmacokinetic; PD, pharmacodynamics; QTc, corrected QT interval; ms, milliseconds; nM, nanomolar; C_max_, maximum plasma concentration; ss, steady state; C_ss_, concentration at steady state; 90% CI, 90% confidence interval; SD, single dose; BID, twice daily; RPED, retinal pigment epithelial detachment; – not available; ∗ Included patients who received doses other than the full dose for NSCLC or dose is unknown. References can be found in the supplementary.

### QTc prolongation

**Fig. 3:**
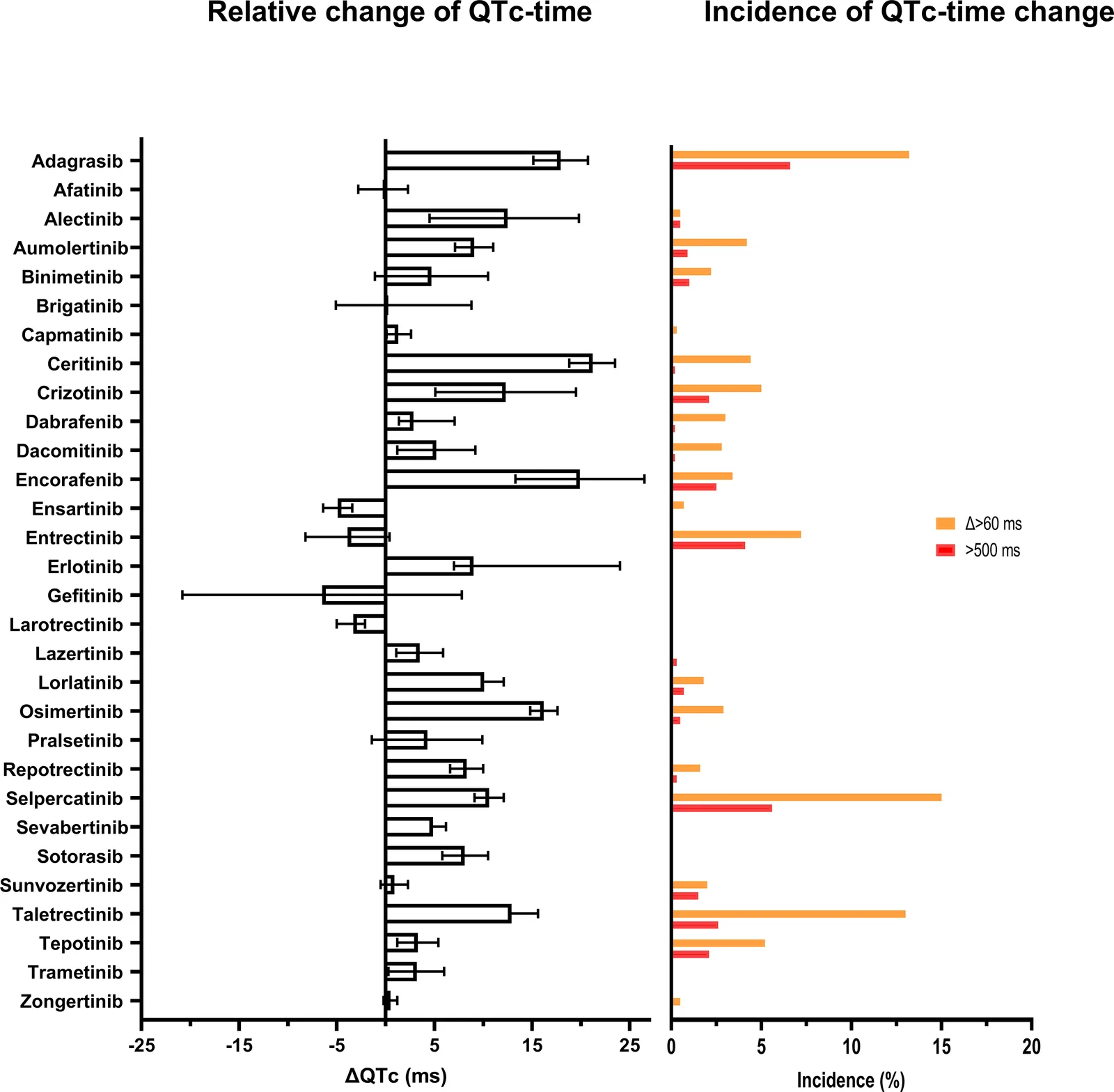
**Corrected QT interval (QTc).** Change in corrected QTc (mean or median ΔQTc, in milliseconds (ms)) from baseline (left) and incidence (%) of QTc prolongation with >60 ms from baseline in orange and a QTc of >500 ms during treatment in red (right), as reported in the regulatory documents. If the data are unavailable, neither a bar nor a value is displayed. For brigatinib, dabrafenib, sevabertinib and sunvozertinib, the presented data demonstrates a dosage exceeding the standard dose. The data for brigatinib and erlotinib are presented as a range of changes in the QTc interval.

Corrected QT interval (QTc) interval prolongation is a class effect of SMIs, though its magnitude and clinical significance varies between agents (Fig. 2 and Table 3). Especially adagrasib, ceritinib, crizotinib, encorafenib, osimertinib and taletrectinib are associated with relatively large median QTc increases from baseline (>10 ms; ms), see Fig. 3.^14^^,^^15^ This effect appears to be exposure-dependent for the majority of SMIs, based on pharmacokinetic data and available dose recommendations outlined in the regulatory documents.^14^^,^^15^ Thus, drug interactions that increase SMI exposure may elevate the risk of QTc prolongation associated with SMIs. The incidence of clinically significant QTc prolongations (QTc increase >60 ms from baseline or QTc >500 ms) is reported to be the highest for adagrasib, encorafenib, and taletrectinib, entrectinib and selpercatinib, see Figs. 3.^14^^,^^15^ These data should be interpreted in the context that the contribution of other QTc-prolonging factors, such as concomitant medication, remains unreported in data from regulatory documents. Conversely, afatinib, alectinib, binimetinib, brigatinib, capmatinib, gefitinib, lazertinib, sevabertinib and zongertinib have not demonstrated clinically meaningful QTc prolongations in either pivotal trials or secondary pharmacology assessments.^14^ Conflicting data is presented for lorlatinib (both QTc shortening and prolongation), though QTc prolongation is in line with the class effect observed with SMIs.^14^^,^^15^

Overall, although QTc prolongation is a common safety concern among SMIs, its risk profile is agent-specific and requires individualised monitoring and management strategies based on drug exposure, comorbidities, and concomitant QTc-prolonging medication. Dose interruptions, dose modifications or discontinuation upon QTc >500 ms or in the presence of arrhythmic symptoms may be necessary.^14^^,^^30^

### Pneumonitis

Pneumonitis, as part of interstitial lung disease (ILD), is a non-infectious inflammation of the pulmonary parenchyma, and a potentially life-threatening adverse event.^14^^,^^31^ It is associated with the majority of SMIs, see Table 3. Although the overall incidence remains low, comparative data from clinical trials indicate that the incidence of pneumonitis varies across SMIs. The incidence of pneumonitis was highest among patients treated with brigatinib, with 9% experiencing all-grade events and 4–6% experiencing grade ≥3 severity, followed by pralsetinib with ∼12% and 3% of patients affected, respectively, including one fatal case.^14^^,^^15^ Other SMIs with a relatively high incidence of pneumonitis and ILD are adagrasib, capmatinib, ensartinib and sunvozertinib with all-grade events reported in 5–6% of patients and grade ≥3 events in 2–4%, including one fatal case reported for adagrasib and capmatinib.^14^^,^^15^ A positive correlation between drug exposure and ILD was established for gefitinib.^14^^,^^32^ Brigatinib is also associated with an increased, exposure-related risk, particularly of early-onset pneumonitis, with the majority of events being severe and four fatal outcomes. Events typically occurred within the first week of treatment, in contrast to the 1–3-month onset observed with other SMIs. Therefore, a dose titration was implemented, starting at 90 mg once daily for 7 days, with escalation to 180 mg once daily in the absence of pneumonitis-/ILD-like symptoms.^14^ For other SMIs, the incidence of all-grade pneumonitis and ILD were reported in ∼1–3% of patients.^14^

Emerging evidence from multiple clinical studies suggests that ethnicity is also an independent risk factor for developing pneumonitis. Asian populations may exhibit an increased susceptibility to SMI-associated ILD.^14^^,^^31^, ^32^, ^33^, ^34^, ^35^, ^36^, ^37^ This has been consistently observed in Japanese patients with several SMIs, including afatinib, crizotinib, brigatinib, gefitinib, and osimertinib.^14^^,^^32^, ^33^, ^34^, ^35^, ^36^, ^37^ Notably, this disparity is most pronounced for gefitinib and osimertinib, with all-grade ILD reported in up to 15% and 17% of Japanese patients, respectively.^14^^,^^32^^,^^34^

In the management of pneumonitis or ILD, regulatory documents often recommend permanent discontinuation of the SMI.^14^ Data on rechallenge following pneumonitis are scarce. In one study, in which patients were rechallenged with EGFR-SMIs after osimertinib-related pneumonitis, the 12-month recurrence rate was 50% when rechallenged with osimertinib and 15% with alternative EGFR inhibitors (afatinib, erlotinib and gefitinib). In addition to the type EGFR-SMI, the onset of pneumonitis within 60 days of initial osimertinib administration was also significantly associated with recurrence.^38^ In contrast, a recent study with a relatively small sample size reported no recurrence of pneumonitis following rechallenge with gefitinib, erlotinib, or afatinib.^39^

These data underscore the agent-specific variability in the risk of pneumonitis across SMIs, with reported incidence rates as high as 37%. A higher incidence of ILD has been observed with EGFR- and anaplastic lymphoma kinase (ALK) inhibitors. Furthermore, ethnic background, i.e. Japanese, influences susceptibility to SMI-induced pneumonitis. When considering rechallenge with SMIs after pneumonitis, a careful, individualised risk–benefit assessment is essential given the high risk of recurrence.

### Weight gain

Certain SMIs exhibit treatment-emergent (serious) weight gain—an adverse effect with potential metabolic and cardiovascular implications.^25^^,^^40^ Clinically significant weight gain has been primarily associated with neurotrophic tropomyosin receptor kinase (NTRK) and ALK inhibitors, typically manifesting within the first months of therapy.

In NTRK inhibitors, weight gain is reported in 53% of patients treated with potent NTRK-targeted agents, with 8% experiencing grade 3 weight gain.^41^ This effect likely reflects on-target toxicity via tropomyosin receptor kinase B (TRKB) inhibition, a neurotrophin receptor involved in hypothalamic regulation of appetite and energy balance.^14^^,^^42^ Entrectinib, which inhibits both TRKB and ALK, has been associated with the highest incidence: ∼66% of patients experienced weight gain, of which 28% developed grade ≥3.^14^ The more pronounced weight gain observed with entrectinib might reflect a stronger potency of NTRK inhibition.^14^^,^^15^ Lower incidences were observed with larotrectinib and repotrectinib, ranging from 12% to 15% for all-grade events and from 2% to 6% for grade ≥3 events.^14^ For repotrectinib, a median weight increase of ∼1 kg from baseline to the end of treatment was observed, with the majority of weight gain occurring during the early treatment cycles.^14^ Taletrectinib, a selective ROS proto-oncogene 1 inhibitor with potential for NTRK inhibition, exhibited a contradictory risk of weight gain, since both weight gain and decrease were observed in ∼11–14% of patients.^15^

Among ALK inhibitors, the highest rates of all-grade weight gain have been reported with lorlatinib (up to 81%) and alectinib (up to 50%).^14^^,^^15^ Grade ≥3 weight gain (defined as ≥20% increase from baseline) occurred in up to 23% of patients receiving lorlatinib.^43^ In a prospective study, lorlatinib treatment led to a median weight increase of 4.5 kg (6.5%) from baseline, with 35% of patients transitioning from a normal to overweight or obese BMI category.^44^ Similarly, alectinib was associated with a mean increase of 9 cm in waist circumference over one year, and a 40% rise in abdominal obesity prevalence.^25^ In a retrospective analysis, significantly higher rates of grade 2–3 weight gain were reported with lorlatinib and alectinib compared to brigatinib and crizotinib.^45^ Moreover, lorlatinib was associated with a more persistent increase in body weight over time, showing a 8% change from baseline within the first six months of treatment, compared to 3% for alectinib, 1.6% for brigatinib, and 0.1% for crizotinib.^45^ No clinically significant correlation was observed between weight gain and plasma exposure levels of alectinib nor lorlatinib.^14^^,^^46^

Weight gain has also been observed with other targeted therapies with all-grade incidence rates similar to larotrectinib and repotrectinib. These SMIs include capmatinib, encorafenib plus binimetinib and trametinib plus dabrafenib.^14^ In contrast, regulatory documents report no clinically meaningful changes in body weight or body mass index in clinical trials for tepotinib and sotorasib; however, the adequacy of weight gain assessment in these studies remains unclear.^14^ For other SMIs, weight gain has not been explicitly reported, either because this adverse effect is absent or because of insufficient detection and reporting. This underscores the need for a systematic evaluation of weight gain in clinical trials involving SMIs.

Given the potential metabolic and cardiovascular consequences of weight gain, patients should be counselled and routine monitoring of body weight is warranted, particularly for agents known to be associated with weight gain. Early lifestyle interventions, including dietary modification and physical activity, remain the cornerstone of care.^47^ This may be challenging for SMIs such as alectinib, requiring co-administration with high-fat meals to ensure adequate drug exposure. Pharmacologic strategies including glucagon-like peptide-1 receptor agonists may also be necessary to mitigate weight gain, particularly in cases where lifestyle interventions are insufficient.^48^ However, potential drug–drug interactions must be carefully evaluated when introducing these agents. For example, a recent study demonstrated a 32% reduction in alectinib exposure when combined with semaglutide.^49^

### Ocular toxicity

Visual disturbances are frequently reported among various SMIs.^14^ More pronounced ocular surface toxicities, including keratitis and conjunctivitis, are predominantly observed with EGFR inhibitors, reflecting on-target inhibition of EGFR expressed in basal epithelial cells of the ocular surface, see Table 3.^14^^,^^50^^,^^51^ EGFR plays a pivotal role in corneal epithelial maintenance and lacrimal gland function, and its inhibition may disrupt epithelial turnover and tear production, leading to barrier dysfunction, dry eyes, and heightened risk of keratitis.^50^, ^51^, ^52^ All-grade keratitis is observed in 1–3% of patients treated with EGFR inhibitors.^14^^,^^15^ A recent study found an overall incidence of ∼2.5% for new-onset keratitis among patients with NSCLC treated with EGFR SMIs.^51^ Notably, second- and third generation EGFR inhibitors are associated with a higher incidence and severity of ocular adverse events compared to first-generation agents, potentially due to an enhanced selectivity for EGFR.^51^ Conjunctivitis has also been reported with EGFR inhibitors, with dacomitinib showing the highest incidence around 24%, predominantly of mild severity, followed by erlotinib at 12%. Lower rates of ∼3% are observed with aumolertinib, gefitinib and osimertinib.^14^ In human epidermal growth factor receptor 2 and NTRK inhibitors, the reported incidence of conjunctivitis was ∼3.5% (sevabertinib) and ∼1% (repotrectinib and taletrectinib).^14^^,^^15^ For other SMIs, such as brigatinib and crizotinib, keratitis and conjunctivitis have not been reported although warnings for vision disorders are included in regulatory documents.^14^

B-Raf proto-oncogene (BRAF)/mesenchymal–epithelial transition (MEK) inhibitors demonstrate different ocular toxicity, in particular uveitis and retinal pigment epithelial detachment. The latter has mainly been seen with encorafenib plus binimetinib, with all-grade events reported in ∼30% of patients, and grade ≥3 events occurring in ∼2%.^14^ Additionally, uveitis incidence ranged from 0.5% to 4.4%, with severe cases in <1%.^14^^,^^53^ Dabrafenib monotherapy and its co-administration with trametinib have been associated with a lower incidence of uveitis (<1%). Cases were generally manageable with topical corticosteroids without necessitating permanent treatment discontinuation.^14^

In clinical practice, the management of SMI-associated ocular toxicities typically involves temporary interruption, followed by dose modifications or permanent discontinuation of SMI therapy, depending on the severity of symptoms, along with prompt referral for ophthalmologic assessment, and discontinuation of contact lenses use when applicable.^14^^,^^15^ For uveitis, short-term administration of topical corticosteroids may be considered to mitigate inflammation and promote epithelial recovery, although their use warrants caution due to the risk of elevated intraocular pressure.^14^^,^^54^

### Neurological toxicity

Central nervous system (CNS) toxicity has been frequently observed with several SMIs; often reported as headache and dizziness.^14^ Cognitive disorders are mainly associated with capmatinib, entrectinib, lorlatinib and repotrectinib, with all-grade incidences ranging from 22 to 32% and grade ≥3 reported in 1–3.5%. Symptoms may include depression, mental slowing, amnesia, delirium, and dementia.^14^^,^^43^ Lorlatinib has also been associated with suicidal ideation.^15^ Lazertinib and larotrectinib are associated with paraesthesia specifically, with an incidence of 34% and 10%, respectively, and grade ≥3 in ∼2%.^14^ This effect was found to be dose-dependent for lazertinib.^14^ Lorlatinib shows the highest incidences of peripheral neuropathy, followed by brigatinib, repotrectinib and taletrectinib. All-grade toxicity was reported in 17–44% of patients and grade ≥3 in 0.3–3%.^14^ Neuropathy in general was reported in 25% of patients treated with crizotinib, while encorafenib combined with binimetinib, and entrectinib show lower rates (11–13%).^14^^,^^43^ Notably, peripheral neuropathy was reported in 8.5% of U.S. patients treated with pralsetinib, compared to 2.3% in European patients at the same approved dose.^14^^,^^15^

CNS toxicity does not consistently correlate with cerebral penetration. Aumolertinib and osimertinib, for instance, effectively penetrates the brain, but CNS toxicity remains absent.^14^ Furthermore, the cerebral penetration of encorafenib, erlotinib and repotrectinib is presumed to be limited, as these are substrates of the drug efflux transporter ABCB1.^14^ ABCB1-inhibition may theoretically enhance cerebral penetration and increase the risk of CNS toxicity, but this has not been clinically confirmed.^14^^,^^55^ Neurological toxicity likely reflects on-target toxicity. For example, capmatinib and crizotinib may induce neurotoxicity due to mesenchymal–epithelial transition expression in neurons and other brain-resident cells including astrocytes, oligodendrocytes, and microglia.^14^ Similarly, NTRK is highly expressed in neuronal tissue and plays a critical role in the development and function of both the central and peripheral nervous systems, suggesting a plausible mechanism for the neurological toxicity of entrectinib and repotrectinib.^14^

Management of neurological toxicity involves temporary interruption of SMI therapy until symptoms improve to grade ≤1.^14^ Dose modifications should be guided by the severity of toxicity and clinical response. In patients with peripheral neuropathy, treatment with gabapentin, duloxetine, or other appropriate neuropathic pain medications may be considered.^56^^,^^57^ Persistent cognitive impairment despite temporary treatment interruption and dose reduction warrants referral to a neurologist or psychiatrist to investigate underlying causes and to consider appropriate pharmacological management, including antipsychotic or antidepressant therapy when indicated.^14^^,^^56^^,^^57^ In case of severe and persistent toxicity, permanent discontinuation of SMI therapy is recommended.^14^

### Pyrexia

Non-infectious pyrexia, defined as elevated body temperature of ≥38 °C in the absence of a clinical or microbial infection, is a common adverse event specifically associated with BRAF inhibitors.^14^ Central thermoregulatory pathways mediated by prostaglandin E2, toll-like receptor-driven inflammatory activation, and cytokine upregulation are considered to contribute to this adverse effect.^14^ A recent study reported non-infectious pyrexia in 39% of patients treated with dabrafenib plus trametinib, with 16% requiring hospitalization or experiencing grade ≥2 adverse events, including dehydration, hypotension, renal dysfunction, confusion or vomiting.^58^ For encorafenib and binimetinib, pyrexia was observed in ∼18% of patients, with grade ≥3 events in 4%. However, the majority of these events were observed after ≥6 months of treatment, possibly reflecting the inclusion of pyrexia events secondary to underlying infections.^14^ Pyrexia typically emerges within the first month, resolves within five days and is correlated with drug exposure.^14^

Therapy should be temporarily interrupted if body temperature reaches ≥38.5 °C, or at the first symptom of recurrence, provided that an underlying infection can be excluded. Antipyretics, such as paracetamol or nonsteroidal anti-inflammatory drugs (NSAIDs), should be administered, and oral corticosteroids may be considered if antipyretic treatment is inadequate or if the fever is accompanied by severe symptoms. Once the fever subsides, therapy can be resumed, with a dose reduction recommended upon rechallenge in severe cases.^14^

### Mucositis

Gastrointestinal toxicity is common for SMIs. For instance, stomatitis, i.e. oral mucositis, is markedly prevalent for EGFR inhibitors, possibly reflecting a class effect. Incidence rates of stomatitis vary considerably, ranging from 9 to 17% for all-grade toxicity with first-generation EGFR inhibitors gefinitib and erlotinib, to substantially higher rates of ≥70% with second-generation inhibitors afatinib and dacomitinib. Grade ≥3 events are most frequently observed with afatinib, affecting nearly 9% of patients, followed by dacomitinib at 5%.^14^ Stomatitis was reported in up to 43% of patients receiving lazertinib and amivantamab.^14^ Aumolertinib and osimertinib, however, demonstrate a lower incidence of ∼12%, all classified as grade 1–2.^14^ The higher rates of mucositis observed with second-generation EGFR inhibitors are likely related to their irreversible targeting of wild-type EGFR, leading to more pronounced disruption of epithelial homeostasis than that seen with reversible first-generation inhibitors or mutant-selective third-generation agents with reduced activity against wild-type EGFR.^59^ Sevabertinib, similar to EGFR inhibitors, causes stomatitis in ∼30% of patients, with ∼1.5% experiencing grade ≥3 events. Stomatitis has also been reported with brigatinib, ceritinib, dabrafenib plus trametinib, pralsetinib and zongertinib, with all-grade events reported in 6–16% of patients.^14^ For taletrectinib, only a few cases were reported.^15^ Stomatitis typically manifests within the first two weeks of treatment and appears to be exposure related.^14^^,^^15^ A dose escalation protocol was implemented solely for afatinib, where the dose may be increased to 50 mg after the initial three-week treatment cycle, contingent upon the absence of gastrointestinal toxicity.^14^

Colitis manifests with a lower incidence compared to stomatitis, and is particularly associated with BRAF/MEK inhibition. Across clinical trials, encorafenib plus binimetinib showed all-grade colitis in 2–6% of patients, and grade ≥3 events in up to 3% of patients.^14^ For dabrafenib plus trametinib, colitis was found in ∼2% of patients, with grade ≥3 events occurring in <1%.^14^ Apart from the BRAF/MEK inhibitors, colitis was rare, with only a few cases reported for adagrasib, crizotinib, pralsetinib, repotrectinib and zongertinib.^14^^,^^15^

In clinical practice, the management of SMI-related mucositis mainly involves maintaining adequate oral hygiene, topical and systemic pain management, and ensuring sufficient nutritional support.^5^^,^^60^ For grade 2 or 3 stomatitis, treatment cessation and/or dose reduction is recommended.^5^ As evidence-based guidelines for SMI-induced colitis are currently lacking, discontinuation of the offending SMI remains the cornerstone of management.^61^

### Hepatotoxicity

Hepatotoxicity is a well-recognised adverse effect associated with the majority of SMIs used for NSCLC, and often necessitates dose interruption, reduction, or permanent discontinuation of treatment.^14^^,^^15^ An alternative strategy, intra-class switching between SMIs due to hepatotoxicity, has been addressed in some studies involving gefitinib and erlotinib, as well as sotorasib and adagrasib. In two studies, patients with disease control on gefitinib who developed hepatotoxicity were subsequently switched to erlotinib. Among these, only a limited number of patients experienced mild hepatotoxicity during erlotinib treatment, indicating a potentially more favourable hepatic safety profile for erlotinib.^62^^,^^63^ Similarly, a successful switch from sotorasib to adagrasib was observed in five patients, with no recurrence of hepatotoxicity.^64^ These findings may indicate distinct mechanisms underlying hepatic injury.^65^

## Conclusions

SMIs are often integrated into the treatment of oncogene-driven NSCLC, posing significant challenges of pharmacokinetic interactions and the management of adverse effects. Food intake, gastric acid-suppressing agents, and concomitant use of CYP-enzymes and drug transporter modulators may substantially affect SMI exposure and should therefore be assessed during treatment. In addition, awareness of class-specific toxicities is essential to ensure safe and effective therapy. Future studies should aim to further elucidate pharmacokinetic–pharmacodynamic associations, as such data remain limited. Establishing these associations could facilitate personalised dosing strategies to minimise toxicity and optimise efficacy.

## Contributors

LMGH, GDMV, DACL, RHJM and FGAJ developed the concept and design for this review. LMGH, GDMV, and FGAJ collected and assembled the data. LMGH, GDMV and DACL verified the data. LMGH led the writing. GDMV, DACL, AKLR, RHNvS, ACD, KT, RHJM and FGAJ were responsible for critical revisions. All authors contributed to data analysis and interpretation, creation of figures and tables, and preparation of the report for publication and they approved the final version of the manuscript.

## Declaration of interests

GDMV received funding with an unrestricted research grant by Pfizer (paid to the institution). ACD has received funding from Dutch Cancer Society, HANART and GIDS (all paid to the institution), consulting fees from Amgen, Bayer, Boehringer Ingelheim, Roche, Johnson&Johnson, Astra Zeneca, Pfizer, BMS, MSD, BeOne, SystImmune and Mirati (all paid to the institution), speaker fees from Johnson&Johnson, Astra Zeneca, Eli Lilly and BeOne (all paid to the institution), fees for Data Safety Monitoring Board/Advisory Board from Roche, Eli Lilly, Boehringer Ingelheim and Amgen (all paid to the institution), and has an unpaid leadership role as past-chair EORTC Lung Cancer Group and co-chair Scientific Chair Council EORTC. KT received funding from PREWAPHARM (Prevent Water Pollution by Pharmaceuticals, 2025–2028, payment to the institution), Happy Patient Project funded by the European Commision (payment to the institution), member of the Data Safety Monitoring Board for the EPABID-study (GGZ Drenthe, unpaid). RHJM received unrestricted grants for investigator-initiated research from Astellas, Bayer, Boehringer Ingelheim, Cristal Therapeutics, Deuter Oncology, Nordic Pharma, Novartis, Pfizer, Roche, Sanofi and Servier (all paid to the institution). This funding was not related to the topics discussed in the current review. LMGH, DACL, AKLR, RHNvS and FGAJ declare no potential competing interests.
