# Supplementary material for: Pharmacological considerations for prescribing of small molecule inhibitors in patients with non-small cell lung cancer
Source: eClinicalMedicine. 2026 Sep 15;100:104199. doi: 10.1016/j.eclinm.2026.104199 (PMC13595012; doi:10.1016/j.eclinm.2026.104199)
Supplement: Supplementary file 1 [file mmc1.docx]

**Table of contents**

[Embase search	3](#_Toc238064071)

[PubMed search	4](#_Toc238064072)

[References Table 1 and 3	5](#_Toc238064073)

## Embase search

(('pharmacokinetics'/mj/exp OR 'pharmacokinetic parameters'/mj/exp OR pharmacokinetic* OR 'pharmaco kinetic*' OR toxicokinetic* OR pk OR 'drug interaction' OR 'interact*' OR 'relation*') AND ('herbaceous agent'/syn OR 'herb'/syn OR 'food'/syn OR 'food intake'/syn OR 'drug transport'/syn OR 'abc transporter subfamily b'/syn OR 'abc transporter'/syn OR 'effusion'/syn OR 'cytochrome p450'/syn OR 'proton pump inhibitor'/syn OR 'solubility'/syn) AND (((interact* OR relation* OR pharmacokinetic* OR 'pharmaco kinetic' OR toxicokinetic* OR pk OR 'pharmacokinetic parameters') NEAR/6 ('herbaceous agent' OR 'herb' OR 'food' OR 'food intake' OR 'drug transport' OR 'abc transporter subfamily b' OR 'abc transporter' OR 'effusion' OR 'cytochrome p450' OR 'proton pump inhibitor' OR 'solubility')):exp,ab,ti,kw)) OR (('pharmacodynamics'/mj/exp OR 'pharmacodynamic parameters'/mj/exp OR 'drug induced disease'/syn OR 'adverse drug reaction'/syn OR toxicodynamic*:ab,ti,kw OR pd:ab,ti,kw OR ((exposure* NEAR/6 (effect* OR efficac* OR toxicit* OR adverse* OR respons* OR safet*) NEAR/6 (relation* OR associat* OR analy* OR model*)):ab,ti,kw)) AND ('fever'/syn OR 'pyrex*' OR 'neuropathy'/syn OR 'neurotoxicity'/syn OR 'pneumonia'/syn OR 'qt prolongation'/syn OR 'torsade de pointes'/syn OR obes* OR 'body weight gain'/syn OR 'metabolic disorder'/syn OR adipos* OR hepatotox* OR 'drug-induced liver injury'/syn OR 'ocular toxicity'/syn OR 'eye disease'/syn OR 'retinal toxicity'/syn) AND ((pharmacodynamic* OR 'pharmaco dynamic*' OR toxicodynamic* OR pd OR relation* OR exposure* OR toxicit* OR respons* OR safet*) NEAR/6 (fever OR pyrex* OR neuropath* OR neurotoxic* OR pneumoni* OR 'qt prolongation' OR 'torsade de pointes' OR obes* OR 'weight gain' OR 'metabolic disorder' OR adipos* OR hepatotox* OR 'drug-induced liver injury' OR 'ocular toxicity' OR 'eye disease' OR 'retinal toxicity'))) AND ('non small cell lung cancer':exp,ab,ti,kw OR nsclc:exp,ab,ti,kw OR (((lung OR pulmonar*) NEAR/3 (cancer OR neoplas*)):exp,ab,ti,kw)) AND ('epidermal growth factor receptor kinase inhibitor':de,ab,ti,kw OR 'anaplastic lymphoma kinase inhibitor':de,ab,ti,kw OR 'braf kinase inhibitor':de,ab,ti,kw OR 'mitogen activated protein kinase inhibitor':de,ab,ti,kw OR 'adagrasib':de,ab,ti,kw OR 'afatinib':de,ab,ti,kw OR 'alectinib':de,ab,ti,kw OR 'binimetinib':de,ab,ti,kw OR 'brigatinib':de,ab,ti,kw OR 'capmatinib':de,ab,ti,kw OR 'ceritinib':de,ab,ti,kw OR 'crizotinib':de,ab,ti,kw OR 'dabrafenib':de,ab,ti,kw OR 'dacomitinib':de,ab,ti,kw OR ‘encorafenib’:de,ab,ti,kw OR 'entrectinib':de,ab,ti,kw OR 'erlotinib':de,ab,ti,kw OR 'ensartinib':de,ab,ti,kw OR 'gefitinib':de,ab,ti,kw OR 'larotrectinib':de,ab,ti,kw OR 'lorlatinib':de,ab,ti,kw OR 'lazertinib':de,ab,ti,kw OR 'osimertinib':de,ab,ti,kw OR 'pralsetinib':de,ab,ti,kw OR 'repotrectinib':de,ab,ti,kw OR 'selpercatinib':de,ab,ti,kw OR 'sotorasib':de,ab,ti,kw OR 'sevabertinib':de,ab,ti,kw OR 'sunvozertinib':de,ab,ti,kw OR 'taletrectinib':de,ab,ti,kw OR 'tepotinib':de,ab,ti,kw OR 'trametinib':de,ab,ti,kw OR 'zongertinib':de,ab,ti,kw OR (('epidermal growth factor receptor kinase*' OR 'anaplastic lymphoma kinase*' OR 'braf kinase*' OR 'mitogen activated protein kinase*' OR 'mesenchymal epithelial transition*' OR 'tyrosine receptor kinase*' OR 'tyrosine kinase*' OR egfr OR alk OR braf OR mek OR ros OR met OR ret OR ntrk) NEAR/3 inhibit*)) AND ('clinical study'/exp OR 'cohort analysis'/de OR 'longitudinal study'/de OR 'retrospective study'/de OR 'prospective study'/de OR 'clinical stud*':ab,ti,kw OR trial*:ab,ti,kw OR random*:ab,ti,kw OR cohort*:ab,ti,kw OR longitudinal*:ab,ti,kw OR retrospectiv*:ab,ti,kw OR prospectiv*:ab,ti,kw OR ((('phase 1' OR 'phase i' OR 'phase 2' OR 'phase ii' OR 'phase 3' OR 'phase iii' OR 'phase 4' OR 'phase iv') NEAR/3 stud*):ab,ti,kw)) NOT ([conference abstract]/lim AND [2000-2022]/py) NOT ([animals]/lim NOT [humans]/lim) AND [english]/lim

## PubMed search

[SMI name] AND ((“Drug Interactions”[Mesh] OR "Cytochrome P-450 CYP3A"[Mesh] OR “CYP3A4” OR “CYP450 enzymes” OR "ATP Binding Cassette Transporter, Subfamily B, Member 1"[Mesh] OR "ATP Binding Cassette Transporter, Subfamily G, Member 2"[Mesh] OR "Organic Cation Transporter 2"[Mesh] OR “P-glycoprotein” OR “P-gp” OR “BCRP” OR “OCT2” OR “ABCB1” OR “ABCG2”) OR ("Proton Pump Inhibitors"[Mesh] OR “Proton pump inhibitor” OR “PPI” OR “acid reducing agent” OR “acid reducing”) OR ("Food-Drug Interactions"[Mesh] OR “Food-drug interaction” OR “Food intake” OR “Food effect” OR “high fat meal”) OR (“QT prolongation” OR “QTc prolongation” OR “ECG”) OR ("Peripheral Nervous System Diseases"[Mesh] OR “peripheral neuropathy” OR “neurotoxicity” OR “neuropathy”) OR (“Pneumonitis” OR "Lung Diseases, Interstitial"[Mesh] OR “interstitial lung disease”) OR (“Ocular toxicity” OR "Keratitis"[Mesh] OR "Conjunctivitis"[Mesh] OR "Uveitis"[Mesh] OR "Retinal Detachment"[Mesh] OR “retinal pigment epithelial detachment”) OR (“stomatitis”[Mesh] OR “colitis”[Mesh]) OR (“Weight Gain”[Mesh] OR “weight increase”) OR (“Fever”[Mesh] OR “pyrexia”))

## References Table 1 and 3

I. Food and Drug Administration. FDA-approved drugs: FDA Application Review Files and Labels [Internet]. [Last accessed on 10 November 2025]. Available from: https://www.accessdata.fda.gov/scripts/cder/daf/.
II. European Medicines Agency. European public assessment reports, assessment history, and product information (Rev 1) [Internet]. [Last accessed on 10 November 2025]. Available from: https://www.ema.europa.eu/en/medicines.
III. Veerman GDM, Hurkmans DP, Paats MS, et al. Influence of esomeprazole on the bioavailability of afatinib: A pharmacokinetic cross-over study in patients with non-small cell lung cancer. *Biomed Pharmacother* 2022; **155**: 113695.
IV. Lanser DAC, de Leeuw SP, Oomen-de Hoop E, et al. Influence of Food With Different Fat Concentrations on Alectinib Exposure: A Randomized Crossover Pharmacokinetic Trial. J Natl Compr Canc Netw 2023; **21**(6): 645–51 e1.
V. Morcos PN, Guerini E, Parrott N, et al. Effect of Food and Esomeprazole on the Pharmacokinetics of Alectinib, a Highly Selective ALK Inhibitor, in Healthy Subjects. Clin Pharmacol Drug Dev 2017; **6**(4): 388–97.
VI. Tugnait M, Gupta N, Hanley MJ, et al. Effects of Strong CYP2C8 or CYP3A Inhibition and CYP3A Induction on the Pharmacokinetics of Brigatinib, an Oral Anaplastic Lymphoma Kinase Inhibitor, in Healthy Volunteers. Clin Pharmacol Drug Dev 2020; **9**(2): 214–23.
VII. Cho BC, Kim DW, Bearz A, et al. ASCEND-8: A Randomized Phase 1 Study of Ceritinib, 450 mg or 600 mg, Taken with a Low-Fat Meal versus 750 mg in Fasted State in Patients with Anaplastic Lymphoma Kinase (ALK)-Rearranged Metastatic Non-Small Cell Lung Cancer (NSCLC). J Thorac Oncol 2017; **12**(9): 1357–67.
VIII. Lau YY, Gu W, Lin T, et al. Assessment of drug-drug interaction potential between ceritinib and proton pump inhibitors in healthy subjects and in patients with ALK-positive non-small cell lung cancer. Cancer Chemother Pharmacol 2017; **79**(6): 1119–28.
IX. Lau YY, Gu W, Lin T, Song D, Yu R, Scott JW. Effects of meal type on the oral bioavailability of the ALK inhibitor ceritinib in healthy adult subjects. J Clin Pharmacol 2016; **56**(5): 559–66.
X. Hohmann N, Bozorgmehr F, Christopoulos P, et al. Pharmacoenhancement of Low Crizotinib Plasma Concentrations in Patients with Anaplastic Lymphoma Kinase-Positive Non-Small Cell Lung Cancer using the CYP3A Inhibitor Cobicistat. Clin Transl Sci 2021; **14**(2): 487–91.
XI. Lin S, Nickens DJ, Patel M, Wilner KD, Tan W. Clinical implications of an analysis of pharmacokinetics of crizotinib coadministered with dexamethasone in patients with non-small cell lung cancer. Cancer Chemother Pharmacol 2019; **84**(1): 203–11.
XII. Xu H, O'Gorman M, Matschke K, et al. Evaluation of Proton Pump Inhibitor Esomeprazole on Crizotinib Pharmacokinetics in Healthy Participants. Clin Pharmacol Drug Dev 2022; **11**(1): 34–42.
XIII. Suttle AB, Grossmann KF, Ouellet D, et al. Assessment of the drug interaction potential and single- and repeat-dose pharmacokinetics of the BRAF inhibitor dabrafenib. J Clin Pharmacol 2015; **55**(4): 392–400.
XIV. Tan EY, Pazdirkova M, Taylor AJ, Singh N, Iyer GR. Evaluation of a Low-Fat Low-Calorie Meal on the Relative Bioavailability of Trametinib and Dabrafenib: Results From a Randomized, Open-Label, 2-Part Study in Healthy Participants. *Clin Pharmacol Drug Dev* 2023; **12**(3): 333–42.
XV. Ruiz-Garcia A, Giri N, LaBadie RR, et al. A phase I open-label study to investigate the potential drug-drug interaction between single-dose dacomitinib and steady-state paroxetine in healthy volunteers. *J Clin Pharmacol* 2014; **54**(5): 555–62.
XVI. Hahn E, Chavira R, Wollenberg L, Tan W, Reddy MB. Impact of posaconazole and diltiazem on pharmacokinetics of encorafenib, a BRAF V600 kinase inhibitor for melanoma and colorectal cancer with BRAF mutations. *Clin Transl Sci* 2023; **16**(12): 2675–86.
XVII. Piscitelli J, Hens B, Tomaszewska I, et al. Effect of Food and a Proton-Pump Inhibitor on the Absorption of Encorafenib: An In Vivo-In Vitro-In Silico Approach. *Mol Pharm* 2023; **20**(5): 2589–99.
XVIII. Piscitelli J, Reddy MB, Wollenberg L, et al. Evaluation of the effect of modafinil on the pharmacokinetics of encorafenib and binimetinib in patients with BRAF V600-mutant advanced solid tumors. *Cancer Chemother Pharmacol* 2024; **94**(3): 337–47.
XIX. Meneses-Lorente G, Bentley D, Guerini E, et al. Characterization of the pharmacokinetics of entrectinib and its active M5 metabolite in healthy volunteers and patients with solid tumors. *Invest New Drugs* 2021; **39**(3): 803–11.
XX. Veerman GDM, Hussaarts K, Peric R, et al. Influence of Cow's Milk and Esomeprazole on the Absorption of Erlotinib: A Randomized, Crossover Pharmacokinetic Study in Lung Cancer Patients. *Clin Pharmacokinet* 2021; **60**(1): 69–77.
XXI. Ling J, Fettner S, Lum BL, Riek M, Rakhit A. Effect of food on the pharmacokinetics of erlotinib, an orally active epidermal growth factor receptor tyrosine-kinase inhibitor, in healthy individuals. *Anticancer Drugs* 2008; **19**(2): 209–16.
XXII. van Leeuwen RW, Peric R, Hussaarts KG, et al. Influence of the Acidic Beverage Cola on the Absorption of Erlotinib in Patients With Non-Small-Cell Lung Cancer. *J Clin Oncol* 2016; **34**(12): 1309–14.
XXIII. Boosman RJ, de Gooijer CJ, Groenland SL, et al. Ritonavir-Boosted Exposure of Kinase Inhibitors: an Open Label, Cross-over Pharmacokinetic Proof-of-Concept Trial with Erlotinib. *Pharm Res* 2022; **39**(4): 669–76.
XXIV. Hamilton M, Wolf JL, Drolet DW, et al. The effect of rifampicin, a prototypical CYP3A4 inducer, on erlotinib pharmacokinetics in healthy subjects. *Cancer Chemother Pharmacol* 2014; **73**(3): 613–21.
XXV. Kletzl H, Giraudon M, Ducray PS, Abt M, Hamilton M, Lum BL. Effect of gastric pH on erlotinib pharmacokinetics in healthy individuals: omeprazole and ranitidine. *Anticancer Drugs* 2015; **26**(5): 565–72.
XXVI. Katsuya Y, Fujiwara Y, Sunami K, et al. Comparison of the pharmacokinetics of erlotinib administered in complete fasting and 2 h after a meal in patients with lung cancer. *Cancer Chemother Pharmacol* 2015; **76**(1): 125–32.
XXVII. Rakhit A, Pantze M, Fettner S, et al. The effects of CYP3A4 inhibition on erlotinib pharmacokinetics: computer-based simulation (SimCYP™) predicts in vivo metabolic inhibition. *Eur J Clin Pharmacol* 2008; **64**: 31–41.
XXVIII. Swaisland HC, Ranson M, Smith RP, et al. Pharmacokinetic drug interactions of gefitinib with rifampicin, itraconazole and metoprolol. *Clin Pharmacokinet* 2005; **44**(10): 1067–81.
XXIX. Yokota H, Sato K, Okuda Y, et al. Effects of Histamine 2-receptor Antagonists and Proton Pump Inhibitors on the Pharmacokinetics of Gefitinib in Patients With Non-small-cell Lung Cancer. *Clin Lung Cancer* 2017; **18**(6): e433–e9.
XXX. Chhun S, Verstuyft C, Rizzo-Padoin N, et al. Gefitinib-phenytoin interaction is not correlated with the C-erythromycin breath test in healthy male volunteers. *Br J Clin Pharmacol* 2009; **68**(2): 226–37.
XXXI. Li J, Pithavala YK, Gong J, LaBadie RR, Mfopou JK, Chen J. The Effect of Modafinil on the Safety and Pharmacokinetics of Lorlatinib: A Phase I Study in Healthy Participants. *Clin Pharmacokinet* 2021; **60**(10): 1303–12.
XXXII. Xu H, O'Gorman MT, Nepal S, James LP, Ginman K, Pithavala YK. Phase 1 Study Evaluating the Effects of the Proton Pump Inhibitor Rabeprazole and Food on the Pharmacokinetics of Lorlatinib in Healthy Participants. *Clin Pharmacol Drug Dev* 2021; **10**(11): 1395–404.
XXXIII. Vishwanathan K, Dickinson PA, So K, et al. The effect of itraconazole and rifampicin on the pharmacokinetics of osimertinib. *Br J Clin Pharmacol* 2018; **84**(6): 1156–69.
XXXIV. van Veelen A, Gulikers J, Hendriks LEL, et al. Pharmacokinetic boosting of osimertinib with cobicistat in patients with non-small cell lung cancer: The OSIBOOST trial. *Lung Cancer* 2022; **171**: 97–102.
XXXV. Suchomel J, Agarwal P, Anders D, et al. Impact of P-gp inhibition on systemic exposure of pralsetinib and dosing considerations. *Clin Transl Sci* 2024; **17**(6): e13818.
XXXVI. Cardona P, Strydom N, Houk B. Impact of Acid-Reducing Agents on Sotorasib Pharmacokinetics and Potential Mitigation of the Impact by Coadministration With an Acidic Beverage. *Clin Pharmacol Drug Dev* 2025; **14**(2): 167–76.
XXXVII. Cardona P, Dutta S, Houk B. Effect of Strong CYP3A4 Inhibition, CYP3A4 Induction, and OATP1B1/3 Inhibition on the Pharmacokinetics of a Single Oral Dose of Sotorasib. *Clin Pharmacol Drug Dev* 2024; **13**(7): 810–8.
XXXVIII. Cardona P, Dutta S, Houk B. Impact of a High-Fat Meal on the Pharmacokinetics of Sotorasib, a KRAS G12C Inhibitor. *Clin Pharmacol Drug Dev* 2024; **13**(11): 1219–26.
XXXIX. Papadopoulos KP, Borazanci E, Shaw AT, et al. U.S. Phase I First-in-human Study of Taletrectinib (DS-6051b/AB-106), a ROS1/TRK Inhibitor, in Patients with Advanced Solid Tumors. *Clin Cancer Res* 2020; **26**(18): 4785–94.
XL. Strotmann R, Lupfert C, Krebs-Brown A, et al. Sensitivity of Tepotinib to Inhibitors or Inducers of CYP3A4 and P-Gp: Drug Interaction Studies and Physiologically-Based Pharmacokinetic Analysis. *Clin Transl Sci* 2025; **18**(7): e70273.
XLI. Wolke S, Sadrolhefazi B, Esmaeili H, et al. Nonclinical and Clinical Assessments of an Optimized Tablet Formulation of the Novel HER2-Selective Tyrosine Kinase Inhibitor Zongertinib: Focus on Relative Bioavailability and Impact of Variation of Gastric pH. *Mol Pharm* 2025; **22**(11): 6522–30.
XLII. Morcos PN, Bogman K, Hubeaux S, et al. Effect of alectinib on cardiac electrophysiology: results from intensive electrocardiogram monitoring from the pivotal phase II NP28761 and NP28673 studies. *Cancer Chemother Pharmacol* 2017; **79**(3): 559–68.
XLIII. Niho S, Goto Y, Toyozawa R, et al. Phase II study of brigatinib in patients with ROS1 fusion-positive non-small-cell lung cancer: the Barossa study. *ESMO Open* 2024; **9**(8): 103642.
XLIV. Gemma A, Kusumoto M, Kurihara Y, et al. Interstitial Lung Disease Onset and Its Risk Factors in Japanese Patients With ALK-Positive NSCLC After Treatment With Crizotinib. *J Thorac Oncol* 2019; **14**(4): 672–82.
XLV. Nebot N, Arkenau HT, Infante JR, et al. Evaluation of the effect of dabrafenib and metabolites on QTc interval in patients with BRAF V600-mutant tumours. *Br J Clin Pharmacol* 2018; **84**(4): 764–75.
XLVI. Castillejo Becerra CM, Smith WM, Dalvin LA. Ophthalmic adverse effects of BRAF inhibitors. *Eur J Ophthalmol* 2022: 11206721221132872.
XLVII. Tamura K, Nukiwa T, Gemma A, et al. Real-world treatment of over 1600 Japanese patients with EGFR mutation-positive non-small cell lung cancer with daily afatinib. *Int J Clin Oncol* 2019; **24**(8): 917–26.
XLVIII. Ma KS, Lo JE, Chodosh J, Dana R. New-onset keratitis associated with epidermal growth factor receptor-based targeted therapies in Han Chinese patients with lung cancer: A multi-center cohort study. *Ocul Surf* 2024; **33**: 23–30.
XLIX. Kloth JS, Pagani A, Verboom MC, et al. Incidence and relevance of QTc-interval prolongation caused by tyrosine kinase inhibitors. *Br J Cancer* 2015; **112**(6): 1011–6.
L. Kawata T, Higashimori M, Itoh Y, et al. Gefitinib exposure and occurrence of interstitial lung disease in Japanese patients with non-small-cell lung cancer. *Cancer Chemother Pharmacol* 2019; **83**(5): 849–58.
LI. Chang SC, Chang CY, Chang SJ, et al. Gefitinib-related interstitial lung disease in Taiwanese patients with non-small-cell lung cancer. *Clin Lung Cancer* 2013; **14**(1): 55–61.
LII. Huang PC, Lin CC, Dana R, Ma KS. Epidermal Growth Factor Receptor Inhibitors for Lung Cancer and the Risk of Keratitis. *JAMA Ophthalmol* 2024; **142**(2): 140–5.
LIII. Marinis F, Wu YL, de Castro G, Jr., et al. ASTRIS: a global real-world study of osimertinib in >3000 patients with EGFR T790M positive non-small-cell lung cancer. *Future Oncol* 2019; **15**(26): 3003–14.
LIV. Zhong H, Jiang S, Yao W, et al. Safety of osimertinib in Chinese patients with non-small cell lung cancer: a multi-center, prospective, observational study. *Future Oncol* 2025; **21**(28):3639-3648.
LV. Mensah SA, Ahmad S, Alruwaili W, Raval R, Gonuguntla K, Patel B. Cardiovascular events in EGFR-mutation non-small-cell lung cancer patients on osimertinib. *Eur J Hosp Pharm* 2025; **33**(1):2-6.
LVI. Sakata Y, Saito G, Sakata S, et al. Osimertinib as First-Line Treatment for Patients With Advanced EGFR Mutation-Positive Non-Small Cell Lung Cancer in a Real-World Setting: Updated Overall Survival Data (OSI-FACT-OS). *Clin Lung Cancer* 2025; **26**(7): e623–e31 e3.
LVII. Skoulidis F, Li BT, Hochmair M, et al. Pooled safety analysis and management of sotorasib-related adverse events in KRAS G12C-mutated advanced non-small cell lung cancer. *Oncologist* 2025; **30**(1): oyae356.
LVIII. Thummalapalli R, Bernstein E, Herzberg B, et al. Clinical and Genomic Features of Response and Toxicity to Sotorasib in a Real-World Cohort of Patients With Advanced KRAS G12C-Mutant Non-Small Cell Lung Cancer. *JCO Precis Oncol* 2023; **7**: e2300030.
LIX. Seto T, Nakane S, Ji L, et al. Real-world safety and effectiveness of entrectinib in Japanese patients with ROS1 gene fusion-positive, unresectable, advanced/recurrent non-small cell lung cancer: Post-marketing surveillance. *Lung Cancer* 2025; **203**: 108478.
